# Virulence plasmid pINV as a genetic signature for *Shigella flexneri* phylogeny

**DOI:** 10.1099/mgen.0.000846

**Published:** 2022-06-27

**Authors:** Giulia Pilla, Gabriele Arcari, Christoph M. Tang, Alessandra Carattoli

**Affiliations:** ^1^​ Sir William Dunn School of Pathology, University of Oxford, Oxford, UK; ^2^​ Department of Molecular Medicine, Sapienza University of Rome, Rome, Italy

**Keywords:** MLST, partitioning systems, *Shigella*, TA systems, virulence plasmid pINV, vST

## Abstract

*
Shigella flexneri* is a major health burden in low- and middle-income countries, where it is a leading cause of mortality associated with diarrhoea in children, and shows an increasing incidence among travellers and men having sex with men. Like all *Shigella* spp., *S. flexneri* has evolved from commensal *Escherichia coli* following the acquisition of a large plasmid pINV, which contains genes essential for virulence. Current sequence typing schemes of *Shigella* are based on combinations of chromosomal genetic loci, since pINV-encoded virulence genes are often lost during growth in the laboratory, making these elements inappropriate for sequence typing. By performing comparative analysis of pINVs from *S. flexneri* strains isolated from different geographical regions and belonging to different serotypes, we found that in contrast to plasmid-encoded virulence genes, plasmid maintenance genes are highly stable pINV-encoded elements. For the first time, to our knowledge, we have developed a *S. flexneri* plasmid multilocus sequence typing (pMLST) method based on different combinations of alleles of the *vapBC* and *yacAB* toxin–antitoxin (TA) systems, and the *parAB* partitioning system. This enables typing of *S. flexneri* pINV plasmids into distinct ‘virulence sequence types’ (vSTs). Furthermore, the phylogenies of vST alleles and bacterial host core genomes suggests an intimate co-evolution of pINV with the chromosome of its bacterial host, consistent with previous findings. This work demonstrates the potential of plasmid maintenance loci as genetic characteristics to study as well as to trace the molecular phylogenesis of *S. flexneri* pINV and the phylogenetic relationship of this plasmid with its bacterial host.

## Data Summary

A complete list of the genomes analysed in this study is available from Figshare: PLSDB 2020 _11_19 (figshare.com) [1].

**Impact Statement**

For the first time, to our knowledge, we have developed a plasmid multilocus sequence typing (pMLST) scheme that exploits plasmid maintenance genes to type *Shigella flexneri* pINV plasmids. In particular, by combining different alleles of the *vapBC* and *yacAB* toxin–antitoxin (TA) systems, and the *parAB* partitioning system, we grouped *S. flexneri* pINV in distinct virulence sequence types. We demonstrated that in contrast to pINV-encoded virulence genes, plasmid maintenance genes are highly stable and can be used to study the molecular phylogeny of *S. flexneri* pINV and its relationship with the chromosome of the respective bacterial host. Several other important human pathogens rely on large plasmids for their virulence, including other *Shigella* spp.; therefore, indicating that pMLST schemes based on plasmid maintenance gens might be suitable for other bacterial species.

## Introduction

*Shigella* spp. are a major causative agent of bacillary dysentery in humans, and responsible for approximately 163 million cases and more than 74 000 deaths each year worldwide [2, 3]. The majority of shigellosis is caused by *Shigella flexneri* [4, 5], which is endemic in low- and middle-income countries (LMICs), including those in sub-Saharan Africa and South-East Asia. In settings where there is relative lack of access to clean water and healthcare, inadequate sanitation and poor nutrition combine to facilitate bacterial transmission and disease, particularly affecting children under 5 years and adults over 70 years of age [2, 6].

Genomic studies have shown that each *Shigella* spp., including *S. flexneri *, has evolved independently from *Escherichia coli* between 35 000 and 270 000 years ago. The critical event that led to the emergence of *Shigella* as an important human pathogen was the acquisition of a single genetic element, the virulence plasmid, pINV [7]. This plasmid is a low-copy non-conjugative ~200 kb plasmid, belonging to the IncFII group, that confers on *Shigella* the ability to invade into and manipulate the environment in host cells [3, 8–10]. The main virulence-associated region in pINV is the *ipa–mxi–spa* locus, which is a 30 kb pathogenicity island (PAI) containing genes required for the assembly of a type 3 secretion system (T3SS), chaperones, regulators and effectors [10]. Genes encoding T3SS effectors are also located outside the PAI with some enclosed by insertion sequences (ISs) [10].

Similar to other large plasmids, pINV imposes significant fitness costs on *S. flexneri *, which have not been entirely negated by compensatory mutations on the plasmid and/or host chromosome, despite their co-existence for hundreds of thousands of years. Instead, besides carrying virulence genes, the plasmid also expresses genes that have ensured its continued persistence in *S. flexneri *. These genes include toxin–antitoxin (TA) systems that act as addiction systems, by mediating post-segregational killing of plasmid-free bacteria, and partitioning systems that distribute plasmids between daughter cells prior to division [11]. *S. flexneri* pINV has three functional TA systems, *vapBC*, *ccdAB* and *gmvAT*, and two inactive TA loci, *yacAB* and *orf 0111–0112*, together with two partitioning systems, *parAB* and *stbAB* [11–13]. Another peculiar feature of pINV is the remarkable prevalence of ISs, which account for 53 % of all plasmid genes [10]. The ISs on pINV are critical for the architecture and evolution of the plasmid, and can lead to the emergence of avirulent strains lacking the T3SS PAI [10, 14, 15].

Currently, *S. flexneri* strains are subdivided into 19 different serotypes based on the structure of their O-antigen, which is a component of lipopolysaccharide (LPS) [16]. Several genes that determine the serotype are phage-encoded and can be transferred between strains [17], complicating the use of serotyping for understanding the evolution and epidemiology of this species [18]. *S. flexneri* has also been typed using molecular methods, such as PFGE and/or multilocus sequence typing (MLST), which uses the sequences of chromosomally encoded housekeeping genes [19, 20]. However, these methods have recently been supplanted by genomic approaches [18], which demonstrate that *S. flexneri* isolates fall in two clades, one clustering with *Shigella boydii* and associated with a single serotype (serotype 6) and the other containing representatives of remaining serotypes (1–5, X, Y) [21]. Because of the critical role of pINV in host : pathogen interactions, there have been attempts to perform sequence-based phylogenetic analysis of *S. flexneri* focussed on the pINV-encoded genes, *ipgD*, *mxiA* and *mxiC* [22, 23]. This study yielded similar results, identifying two main sequence forms of pINV, pINV A and pINV B, with pINV A being present in serotype 6 and 6A *S *. *flexneri* strains, while pINV B was found in *S. flexneri* strains belonging to serotypes 1A, 2A, 3A, 3C, 4A and Y, consistent with genomic approaches [22, 23]. However, the genes used by this analysis are located in the T3SS PAI, which is frequently lost in the laboratory due to spontaneous IS-mediated deletions [15], making these pINV-encoded genes unsuitable for broader application.

In this study, we aimed to understand the conservation and phylogenetic relationships of backbone genetic elements of *S. flexneri* pINV with the host chromosome to gain insights into the evolution and adaption of the virulence plasmid to its bacterial host. We identified conserved pINV-encoded genes by performing comparative analysis of pINVs from a collection of 36 fully assembled pINV sequences, as well as 29 whole-genome sequences (WGSs) from different *S. flexneri* strains isolated from different geographical regions and belonging to different serotypes. We found that several *S. flexneri* pINV sequences lost extended regions, including T3SS-related genes, highlighting the unsuitability of the *ipgD–mxiA–mxiC* typing system [22, 23]. However, we found that pINV has a highly conserved core genetic region that is rarely lost. This region contains elements of its replicon, specific TA systems and partitioning systems. We describe a novel plasmid MLST (pMLST) scheme that combines different alleles of the *vapBC* and *yacAB* TA systems, and the *parAB* partitioning system, to type *S. flexneri* pINV plasmids, grouping *S. flexneri* pINV in distinct virulence sequence types (vSTs). The classification of pINV plasmids in vSTs showed striking correlation with the phylogenetic clades defined by analysis of the *S. flexneri* genome, suggesting that pINV, and particularly its backbone, has co-evolved with the chromosome.

## Methods

### *S. flexneri* plasmid sequence typing and phylogenetic analysis

A total of 100 assembled plasmid sequences from *S. flexneri* isolates were downloaded from the database described by Galata *et al*. in 2019 (PLSDB, https://ccb-microbe.cs.uni-saarland.de/plsdb/) on the 28th October 2020 [1]. The Inc group and replicon were identified using PlasmidFinder and pMLST [24]. When not indicated, serotype was predicted from genome sequences, as described elsewhere [25, 26]. Only plasmids carrying an IncFII [F27: A−: B−] replicon with at least one of the three genes *virB*, *virF* and/or *virK*, with an available chromosomal sequence, were included in the genomic study for the development of the pINV typing scheme (Tables 1 and S1, available with the online version of this article). Sequence identity of *vapBC*, *ccdAB*, *gmvAT*, *orf 0111–0112*, *yacAB*, *parAB* and *stbAB* on selected pINVs was identified by blastn searches of the sequences using the corresponding genes in pWR100.

**Table 1.** Plasmids used for the development of the vST scheme List of strains analysed in this study with plasmid and chromosome accession numbers, country of origin, vST alleles and groups. When there is no information about the country of isolation, the country where the strain was sequenced is indicated with an asterisk. Serotypes predicted from genome sequencing are indicated with a plus [25, 26]. nt, Non-typeable;NS, not specified.

Plasmid accession no.	Chromosome accession no.	Strain	Serotype	Country of origin	*vapBC* allele	*parAB* allele	*yacAB* allele	vST group
CP030916.1	CP030915.1	1508	2a_2_	China	1	1	1	**1**
CP050984.1	CP050985.1	FDAARGOS_716	2b_1_^+^	Nigeria				
CP055137.1	CP055138.1	FDAARGOS_690	2b_1_^+^	Nigeria				
CP058591.1	CP058589.1	M2901	2b_1_	Australia				
CP001384.1	CP001383.1	2002017	Fxv	China				
CP007038.1	CP007037.1	G1663	2a_2_	China				
CP012142.1	CP012140.1	1205	4c (X^+^)	China				
CP020087.1	CP020086.1	670	1a	China				
CP020337.1	CP020336.1	1602	4c (X^+^)	China				
CP026794.1	CP026793.1	74-1170	5a_1_	USA*				
CP033511.1	CP033510.1	2016AM-0877	2a_2_	USA*				
CP034933.1	CP034931.1	2013C-3749	3b	USA*				
CP044154.1	CP044152.1	AR-0425	2a_2_	USA*				
CP044157.1	CP044155.1	AR-0424	Y^+^	USA*				
CP044160.1	CP044158.1	AR-0423	2a_2_^+^	USA*				
CP012138.1	CP012137.1	981	2a_2_	China*				
CP020343.1	CP020342.1	439	1a	China				
LR213456.1	LR213455.1	AUSMDU00008332	2a_2_^+^	Australia*				
CP045942.1	CP045941.1	AUSMDU00010535	2a_2_	Australia				
CP054887.1	CP054886.1	FDAARGOS_689	2a_2_^+^	Nigeria				
CP026789.1	CP026788.1	ATCC 29903	2a_2_	ns	1	1	/	**nt**
AF386526.1	AE005674.2	301	2a_2_	China	1	7	1	**2**
NC_024996.1	CP037923.1	M90T	5a	USA	2	2	2	**3**
CP026800.1	CP026799.1	NCTC 9728	5a (5b^+^)	USA*	2	3	2	**4**
CP030773.1	CP020753.1	Y394	1c	Bangladesh	2	6	1	**5**
CP026791.1	CP026792.1	61-4982	4b	USA*	2	/	1	**nt**
CP026771.1	CP026768.1	93-3063	Y_1_	USA*	2	1	1	**6**
CP024476.1	CP024473.1	94-3007	7b (7a^+^)	USA*	6	1	1	**7**
CP034059.1	CP034060.1	FDAARGOS_535	1b^+^	ns				
CP055125.1	CP055124.1	FDAARGOS_714	6	Nigeria	4	4	2	**8**
CP026812.1	CP026811.1	64-5500	6	USA*				
CP024472.1	CP024470.1	71-2783	3a	USA*	3	5	2	**9**
CP026804.1	CP026803.1	89-141	Y^+^	USA*	5	5	2	**10**
LR861786.1	LR861785.1	AUSMDU00021847	3a_1_^+^	Australia*	2	5	2	**11**
LR861789.1	LR861788.1	AUSMDU00022017	3a_1_^+^	Australia*				
LR878366.1	LR878365.1	83	3a_1_^+^	UK				
CP026099.1	CP026098.1	FDAARGOS_74	3a_1_^+^	USA	2	5	3	**12**
CP054891.1	CP054892.1	FDAARGOS_713	3a_1_^+^	Nigeria				

The pINV typing scheme was validated on a total of 29 WGSs of *S. flexneri* clinical strains selected from GenBank. The presence of pINV was confirmed by checking for the presence of an IncFII [F27: A−: B−] replicon and one of the *virB*, *virF* and/or *virK* genes using blastn, and serotype was predicted from the WGSs of tested strains.

### Plasmid alignment using brig

blast Ring Image Generator (brig) was employed to align assembled plasmid sequences and either pWR100 (NC_024996.1) or pSF150801 (CP030916.1) were used as reference sequences. For Fig. 4, the parameter ‘-word size 570’ was used to avoid misalignment of multiple copies of ISs.

### Circos plots

Circos plots [27] were used to represent the distribution of the genes selected for vST alongside the 36 IncFII [F27: A−: B−] pINV plasmids and the scattering of ISs in representatives of the vST groups. The presence and location of the genes were assessed by blastn, and synteny evaluated using Mauve [28]. All analyses were performed at the Galaxy server (https://usegalaxy.eu/).

### Phylogenetic analysis

Two different phylogenetic analyses were performed and compared; one was generated from the mafft alignments of a concatenation of the genes (*parAB*, *yacAB* and *vapBC*) considered during the development of vST using iq-tree. A phylogenetic analysis based on the whole-genome core genes employed sequences annotated using Prokka [29], and the resulting General Feature Formats (GFFs) were analysed to identify the core and accessory genes using Roary v3.11 [30]. The Gubbins algorithm was then used to remove recombining regions and to generate a maximum-likelihood (ML) phylogenetic tree using RAxML [31]. The visualization of the trees, metadata and core and accessory genes was performed using Phandango [32], then adjusted using the open source InkScape software (https://inkscape.org/it/).

### Core-genome MLST (cgMLST)

The EnteroBase *Escherichia */* Shigella* cgMLST scheme (based on 2513 genes) was used. The single-linkage hierarchical clustering pipeline (pHierCC), implemented in the ‘cgMLST V1+HierCC V1’ scheme at the EnteroBase database [33], was used to assign the isolates to 13 different clusters. Specifically, the 13 clusters are defined by their resolution level and range from HC0 (no allelic differences) to HC2350 (genomes with up to 2350 allelic differences). The result is indicated by stable numbers. In two cases, two genomes were equidistant from two clusters from the level HC0 to HC20; we assigned them to the cluster with the smallest HC number.

### Experimental identification of vSTs

As an alternative to genome sequencing, a PCR-based pMLST scheme was developed based on the *vapBC*, *yacAB* and *parAB* operons, targeting the sequence that started from the ATG of the first gene of the operon (i.e. *vapB*, *parA* and *yacA*) to the TGA of the second gene of the operon (i.e. *vapC*, *parB* and *yacB*). Amplification reactions contained 2.5 U *Taq* DNA polymerase (Sigma-Aldrich), 1× PCR buffer, 1 μM primers (Table 2, primers 1 and 2 for each locus), 200 μM dNTPs and 1 μl boiled sample. Boiled DNA obtained from a single colony of an isolate with a known chromosomal sequence but unsequenced pINV plasmid (i.e*. S. flexneri* 2a 2457T) was used as DNA template to test the PCR-based pMLST approach. Amplification was performed with an annealing temperature of 58 °C and an extension time of 2 min and 30 s (Fig. S1).

**Table 2.** Sequences of alleles and primers for vST analysis List of alleles of vST groups with corresponding accession numbers of reference plasmid sequences and SNPs, and primers for amplifying and sequencing alleles.

Allele	Reference accession no.	Allele SNP	Primer
*vapBC1*	CP030916	Reference allele	5′-GCGATACTCATCATAAACGTATATCCC-3′ 5′-GTTGAGCCGGGAGATGATTTC-3′
*vapBC2*	CP037924	A210G	
*vapBC3*	CP024472	G36A; A210G; A322G	
*vapBC4*	CP055125	G36A; A172G; A210G; A322G	
*vapBC5*	CP026804.1	A210G; A322G	
*vapBC6*	CP024476.1	A210G; G242T	
*parAB1*	CP030916.1	Reference allele	5′-GTTAAAATCCTACATAGCACGGAGG-3′ 5′-CGTGAAAGAGGGGGAAGACTATC-3′ 5′-TGCGAATCCTGTTACTTATGTTGG-3′ 5′-CCATCAGTAGTTGGCCCTTATAAATTC-3′
*parAB2*	CP037924.1	G171C; C519T; T1358C	
*parAB3*	CP026800.1	G171C; T1358C	
*parAB4*	CP055125	T1358C; G1703A	
*parAB5*	CP024472	T1358C	
*parAB6*	CP030773.1	C1811T	
*parAB7* *parAB8*	AF386526.1 AAZJOL	T1376C G637T; T1358C	
*yacAB1*	CP030916	Reference allele	5′-GGCATATCCTGGTGACGATATCTG-3′ 5′-ATGGTAGATGTTATTTTCATGTCATACCTTTG-3′
*yacAB2*	CP037924	T175C	
*yacAB3*	CP026099	T175C; A392G	

The amplification products were analysed by Sanger sequencing with the primers indicated in Table 2 (primers 1 and 2 for *vapBC* and *yacAB*, primers 1, 2, 3 and 4 for *parAB*) and the sequences were aligned to the vST allele database using blastn. The genome of strain 2457T was then included in a phylogenetic analysis with 19 genomes belonging to vST1, and one genome representing each of other vST groups, excluding group 8.

### Identification of ISs

ISs were identified using ISfinder [34] and found using blastn following the parameters indicated in Table S2.

## Results

### Comparative analysis of virulence plasmids from *S. flexneri*

A collection of a total of 100 complete circular sequences of *S. flexneri* plasmids was downloaded from the plasmid database described by Galata *et al*. in 2019 [1] (PLSDB, https://ccb-microbe.cs.uni-saarland.de/plsdb/; 28th October 2020; Table S1). Plasmid pWR501 (accession no. AF348706.1) was eliminated as it is identical to pWR100 (NC_024996.1), which were both derived from *S. flexneri* 5a M90T [35]. Among the 99 *S *. *flexneri* plasmids, 57 carried IncF replicons, 39 replicons belong to the IncI1, IncX1, IncN, IncB/O and Col-like types, and 3 were identified as phages. A total of 39 of the 57 IncF-replicon positive plasmids had the FAB formula [F27: A−: B−], which is the same found in the reference *S. flexneri* pINV plasmid, pWR100, and 38 of these plasmids had associated chromosomal sequences (Table S1). These plasmids were selected for further study as *S. flexneri* virulence plasmids (pINV), being positive for at least one of the three virulence genes, *virK* (reference gene ID: 1254304), *virF* (gene ID: 58461125) and *virB* (gene ID 1237991) (Table S1). Of note, these plasmids were from strains of diverse serotype and country of origin, indicating that they do not originate from a single outbreak or clonal expansion (Table 1).

**Fig. 1.**
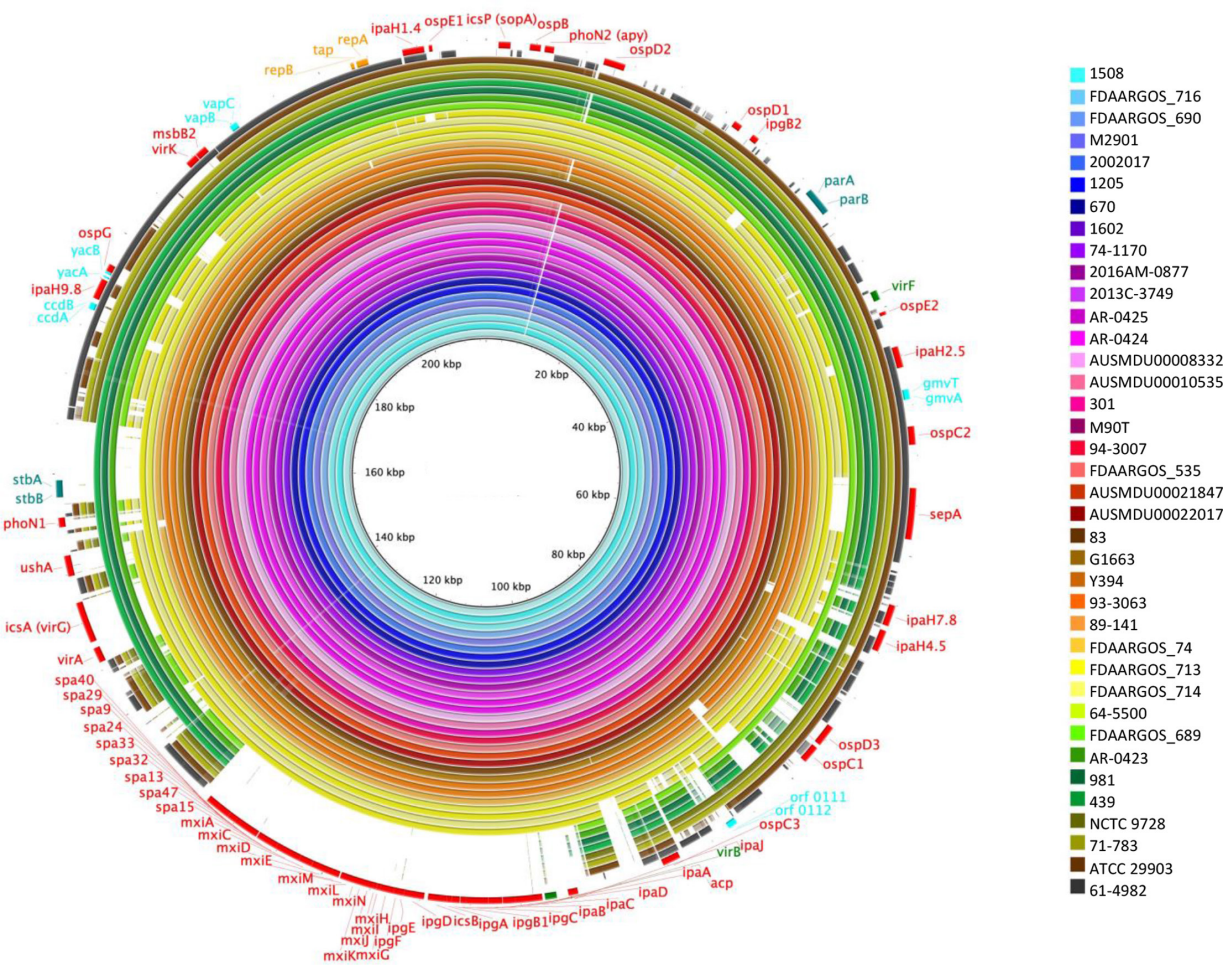
Alignment of *S. flexneri* pINVs showing deletions involving virulence-associated genes. brig 0.95 and blastn v2.2.29 alignment of pINV sequences from *S. flexneri* strains. *S. flexneri* M90T pWR100 (inner black ring, NC_024996.1) is shown as the reference. Features in the external ring have been colour-coded: red for virulence-associated genes, green for transcriptional regulators, teal for partitioning systems, aqua for TA systems, orange for replicon-associated genes. The plasmids analysed are shown in the key and are colour coded.

**Fig. 2.**
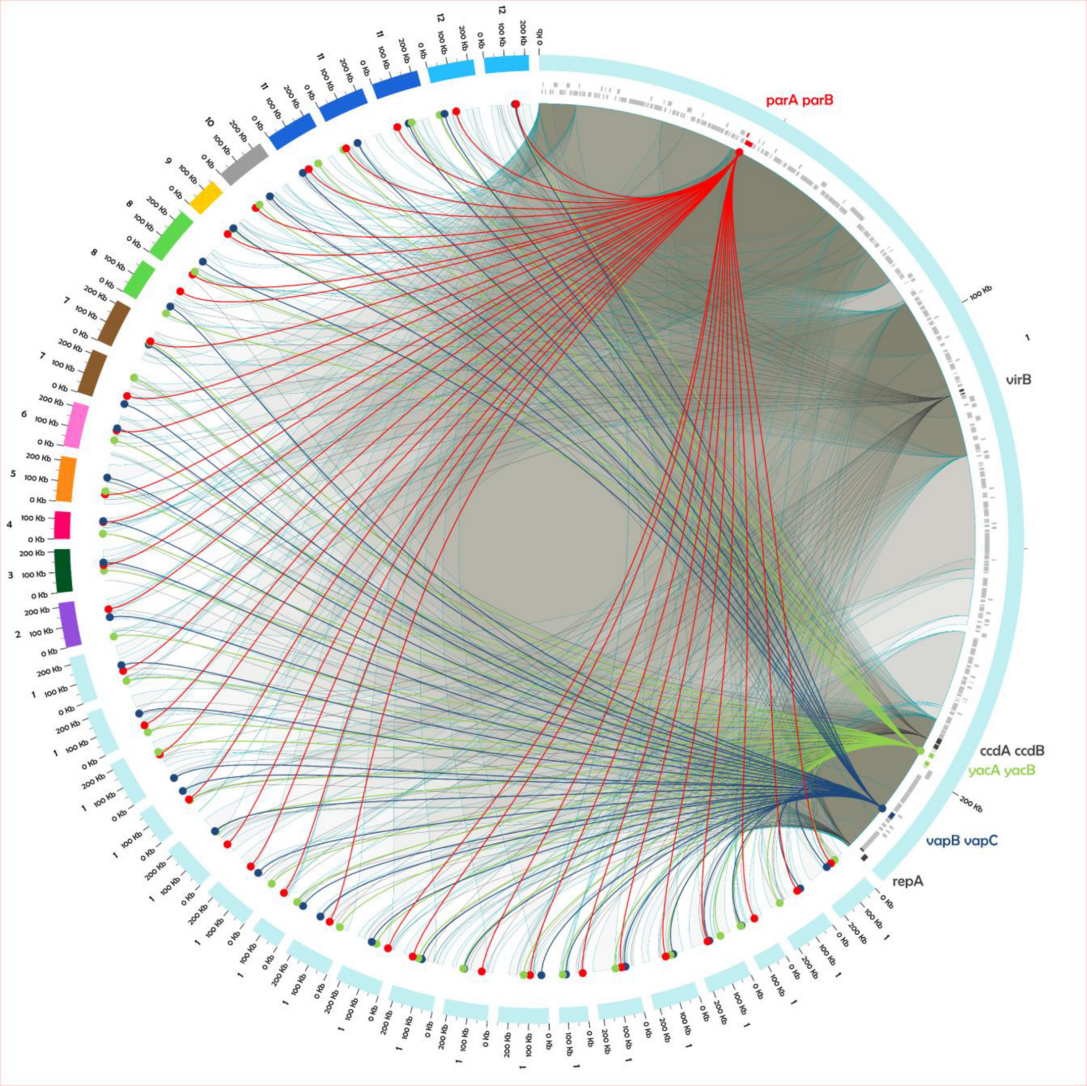
Circos plot of the F27: A−: B− plasmids from the 42 strains analysed in this study. The plasmids are labelled and colour-coded based on their belonging to their vST groups. The reference plasmid for the group 1 (CP030916.1) is located in the upper right of the plot, scaled up 25 times, and with links to related sequences in other plasmids. For instance, the light-grey ribbons outlined in cyan represent the connection between the different regions of the CP030916.1 plasmid and all the other ones. Coloured lines represent the distribution of the genes used to determine the vST (namely *parA* and *parB* in red, *yacA* and *yacB* in green, and *vapB* and *vapC* in blue), while black links indicate other conserved genes. Genes belonging to the CP030916.1 plasmid are indicated in grey or coloured tiles in the row immediately internal to the representation of the plasmid.

The sequences of the 38 selected pINVs were aligned with pWR100 as the reference using brig to identify the conserved regions in the plasmids (Fig. 1). Over the 38 plasmids, 8 showed large deletions involving the T3SS PAI, and 2 plasmids (from strains FDAARGOS_714 and 64–550) had an intact T3SS PAI but had deletions of other virulence genes (i.e*. ospG*, *phoN1*, *sepA*). In contrast, the region encoding plasmid maintenance genes, such as the replicon, functional TA systems *ccdAB*, *yacAB*, *vapBC* and *gmvAT*, and the *parAB* partitioning system*,* showed higher conservation, being present in all but two plasmids (Figs 2 and S2, Table S1); in strains ATCC 29903 and 61-4982 more than 50 % of the plasmid sequence was absent, including *yacAB* and *ccdAB*, and *parAB* (Fig. 1, Tables 1 and S1).

### Plasmid maintenance genes as suitable sequences for *S. flexneri* pINV plasmid typing

The conservation of the *parAB* partitioning system and the *vapBC*, *ccdAB*, *gmvAT* and *yacAB* TA systems in 36 out of 38 *S *. *flexneri* pINVs (Fig. 1) was further assessed by aligning the DNA sequences of these genes with the corresponding genes from pWR100 [35] using blast (Table S1). All strains except for ATCC 29903 and 61-4982 had *vapBC*, *yacAB* and *ccdAB*, and *parAB*, but with variation in their sequences (Figs 2 and S2, Table S1). In contrast, the remaining TA system *orf 0111–0112* and partitioning system *stbAB* were missing in six and nine isolates, respectively (Table S1), confirming that these loci are frequently affected by plasmid rearrangements and, thus, were excluded from our subsequent analysis.

Analysis of sequence identity of *parAB*, *vapBC*, *ccdAB*, *gmvAT* and *yacAB* showed that *ccdAB* and *gmvAT* display less variation compared with the other loci; 32/40 and 29/40 plasmids have identical *ccdAB* and *gmvAT* sequences, respectively, compared to 22/40, 26/40 and 24/40 plasmids for *vapBC* and *yacAB* and *parAB*, respectively (Table S1). Therefore, because of their low discriminatory power, *ccdAB* and *gmvAT* were not included in our analysis. We then analysed the sequence variation in *vapBC*, *parAB* and *yacAB*, and a progressive number was assigned to each unique allele found in each locus, generating an allele database containing a total of 6, 7 and 3 alleles, respectively (Table 2). Sequence type numbers were assigned to each combination of different alleles, grouping the 36 pINV sequences into 12 distinct groups, named vSTs. Because strains ATCC 29903 and 61-4982 lacked *yacAB* and *parAB*, respectively, it was not possible to allocate these strains to a vST (Table 1, Fig. S2).

To further assess the validity of the vST groupings and to detect potential new alleles, 29 WGSs of *S. flexneri* clinical strains were arbitrarily selected from GenBank (Table S3). vST groups were identified by using blastn to align the sequence of *vapBC*, *parAB* and *yacAB* from these strains with the sequences of vST alleles database (Table 2). Most of the selected clinical strains were assigned to vST11 (14 strains), while 9 and 4 strains were identified as vST1 and vST12, respectively; a single strain was associated with vST7 (Table S3). Out of 29 selected WGSs, only one plasmid could not be assigned to one of our pre-existing vSTs, as it contained a novel SNP in *parAB* locus, producing *parAB* allele 8, which was added to the allele database and the plasmid was assigned to vST13 (Table 2).

### Phylogenetic analysis shows co-evolution of pINV virulence plasmid with the chromosome of its respective bacterial host

**Fig. 3.**
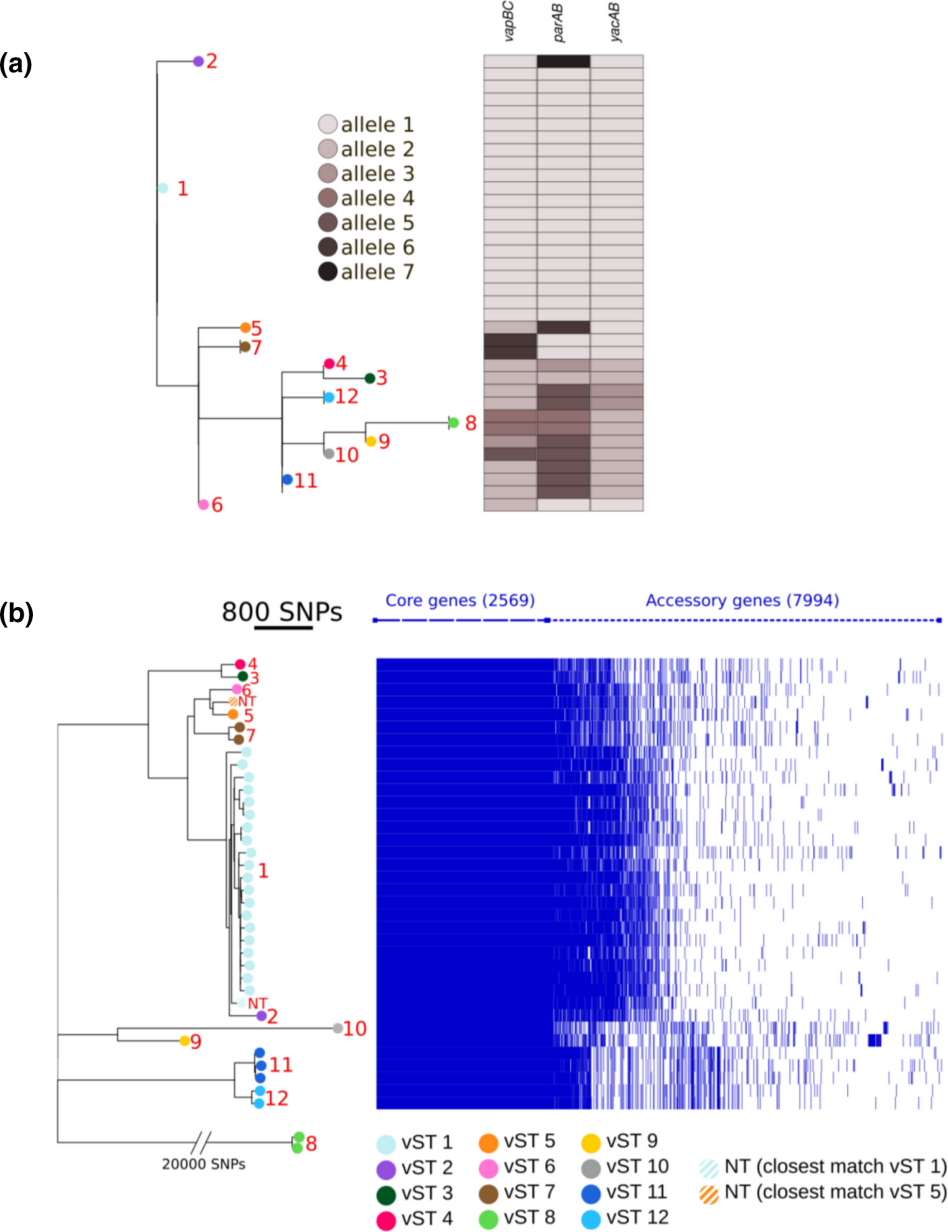
Comparison of the phylogenetic tree of the *S. flexneri* genome with the clustalw-based alignment of *parAB*, *yacAB* and *vapBC*. (a) This shows the tree deriving from the ML iq-tree on mafft alignments of the three loci used to generate the vST (*parAB*, *yacAB* and *vapBC*). Metadata are colour-coded, according to the key on the right, and represent the different allelic combinations that led to the definition of the vST groups. (b) The left side shows an unrooted ML phylogenetic tree based on a concatenated CG (2569 core genes) alignment of 38 *S. flexneri* complete genomes. The legend and red numbers on the right of the tips illustrate the colours assigned to each vST group. The bar on the top left displays the phylogenetic distance between strains in terms of SNPs in the CG. For the sake of clarity group 8, being phylogenetically distant (>20 000 SNPs in the CG) from the other eleven groups, has been hand-inserted in the phylogenetic tree. The middle part represents metadata, each block indicating the assigned vST group. On the right side, each row represents gene content in a given strain, with blue lines representing the presence of a gene. nt, Non-typeable.

We performed ML analysis on the multiple sequence mafft alignment of concatenated *vapBC*, *parAB* and *yacAB* alleles (Fig. 3a), and compared it with an unrooted ML phylogenetic analysis based on the concatenated core genome (CG) of *S. flexneri* containing 2569 core genes (Table 1, Fig. 3b). The phylogenetic analysis of chromosomal CG separated strains into different clades.

Comparative analysis of phylogenetic trees generated by *vapBC*, *parAB* and *yacAB*, and the CG demonstrated that there was a striking concordance between the clades generated by vST groups from pINV plasmid alleles and the clades generated by chromosomal CG analysis of strains. In particular, pINV vST8 belongs to strains that fall in the most distant clade (>20 000 SNPs in the CG), while strains carrying the vST1 pINV are all clustered together in the same clade (<600 SNPs in the CG) (Fig. 3). The chromosome of strains ATCC 29903 and 61-4982 clusters with those of strains carrying vST1 and vST5 plasmids, respectively. The pINVs of these two strains could not be allocated to specific vST groups but their plasmids showed two of three loci identical to vST1 and vST5, respectively (Table 1). This suggested that despite the deletions, their plasmids clustered in the same clades as their chromosomes (Fig. 3b).

**Fig. 4.**
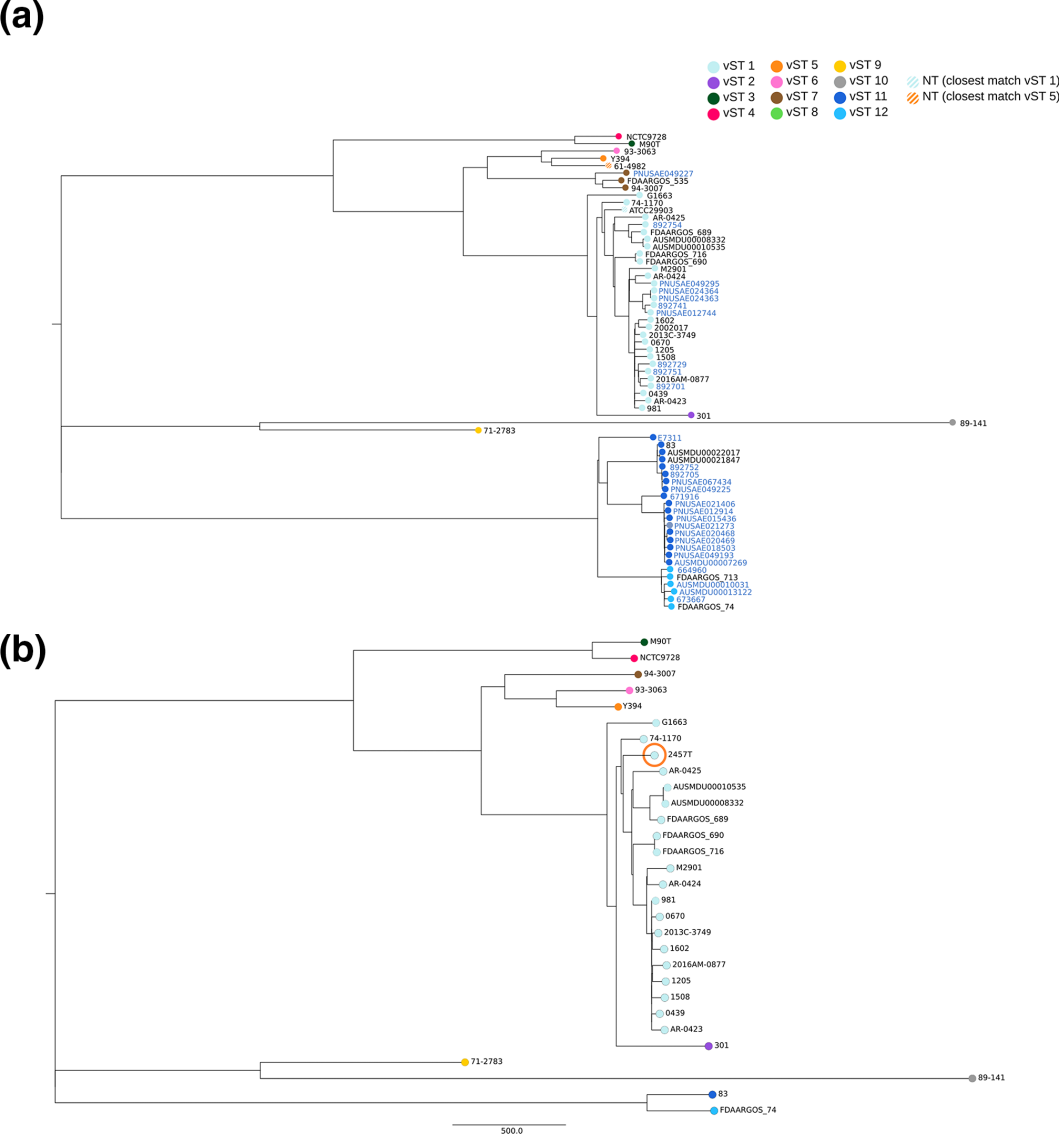
Unrooted ML phylogenetic trees based on alignment of concatenated core genes. (a) A total of 2650 concatenated core genes from 65 *S *. *flexneri* genomes: 36 references (in black, indicated in Table 1, except those assigned to vST8 due to their high phylogenetic distance) compared with 29 incomplete genome sequences from clinical strains downloaded from GenBank (in blue, indicated in Table S3). (b) A total of 3106 concatenated core genes of 29 *S *. *flexneri* genomes downloaded from GenBank, with the 2457T genome (circled in orange) as the reference. A total of 19 genomes belong to vST1, and the remainder total belong to other vST groups with one genome representing each group (with the exception of group 8). nt, Non-typeable

To confirm the association of vST groups with the CG-based phylogenetic clusters, we repeated the same analysis by adding to the ML-phylogenetic analysis the CGs of 29 WGSs of *S. flexneri* clinical strains (Table S3). In this analysis, the two strains belonging to vST8 were not included in the tree due to their high phylogenetic distance from other strains (Fig. 3b). The phylogenetic trees based on vST and on chromosomal CG showed an identical overall structure, with four clusters derived from four distinct primary branches (Fig. 4a). In detail, the ML phylogeneses obtained from WGSs of strains carrying vST11 plasmids (14 strains) showed that all these strains clustered together in a single clade. The same was observed comparing the WGSs of strains carrying plasmids assigned to vST1 (nine strains) or vST12 (four strains) groups. The clusters produced following the phylogenetic analysis of alleles of *vapBC*, *parAB* and *yacAB* coincided with clusters generated by the analysis of SNPs in WGSs of the corresponding strains, except for strains carrying the vST13 plasmid, which fell in the same CG cluster as vST11 strains. In this case, the vST method was more discriminatory than CG analysis, considering that vST13 differs from vST11 by a single SNP in *parAB* genes.

To evaluate the strength of the new vST grouping, we compared this method with the current gold standard method based on the EnteroBase *Escherichia */* Shigella* cgMLST scheme. Out of the total 68 *S *. *flexneri* genomes analysed in this study (Tables 1 and S3), only 43 vST-typeable and 2 vST-nontypeable strains were found in the EnteroBase database and, therefore, could be classified using both the EnteroBase *Escherichia */* Shigella* and cgMLST schemes. We found that the resolution level of the vST scheme is higher than the resolution level of HC400 clustering, but lower when compared to the one of the HC200 clustering (Table S4). In fact, out of 12 of the 13 vSTs, the HC400 clustering was able to identify eight total sequence typing groups and did not discern vST3 from vST4, vST5 from vST6 and vST11 from vST12, while HC200 had a higher discriminatory power, identifying each vST and subdividing vST11 into two groups. Interestingly, one isolate from vST1 (892741) had the same vST11 identifiers up until HC1500, while the vST2 isolate (301) could only be discerned from vST1 at HC100. These results demonstrate that the vST scheme can be a straightforward typing method that does not require genomics and gives information on *S. flexneri* relatedness using as phylogenetic markers the selected plasmid maintenance genes.

To demonstrate the utility of vST grouping, a PCR and Sanger sequencing-based pMLST scheme was also developed to identify vSTs without the need of genomic sequencing of *S. flexneri* strains. PCR primers were designed to amplify and sequence *vapBC*, *parAB* and *yacAB* (Table 2), and used to identify the vST of *S. flexneri* 2a 2457T. For this strain, there is a complete chromosomal sequence available but no information regarding the sequence of pINV (BioProject PRJNA408) [36]. Following Sanger sequencing of the *vapBC*, *parAB* and *yacAB* amplicons, pINV of *S. flexneri* 2a 2457T was assigned to the vST1 group (Fig. 4b, Table 3). Phylogenetic analysis using the CG demonstrated that, consistent with the previous results obtained with the vST approach, *S. flexneri* 2457T CG fell in the same clade with other isolates belonging to vST1 (Fig. 4b).

### pINV plasmids of different vST groups show distinct IS profiles

**Fig. 5.**
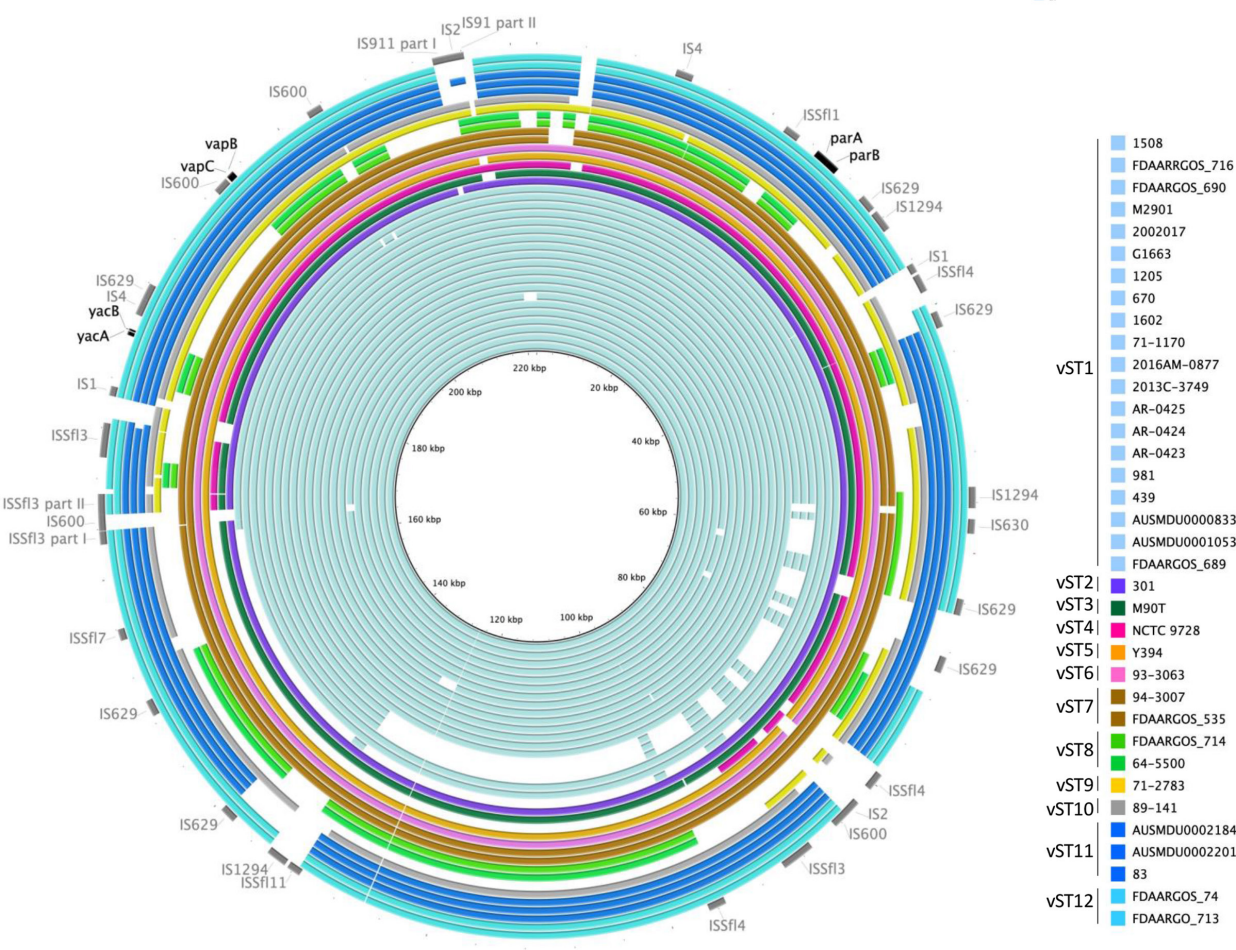
The distribution of ISs differs between the vST groups. Alignment of pINV sequences from *S. flexneri* strains following vST grouping (colour coded as in the key). Image created using brig 0.95 and blastn v2.2.29. *S. flexneri* 1508 pSF1508 (inner black ring) is shown as the reference. In the outer ring, ISs are indicated in grey, and genes used for assigning the vST in black.

As ISs can profoundly influence plasmid dynamics [14], the distribution of ISs within each vST group was investigated. We identified IS loci following alignment of pINV sequences from *S. flexneri* strains belonging to the same vST groups. We found that strains belonging to the same vST group shared almost identical IS profiles on pINV (e.g. vST1, 7, 8, 11 and 12; Figs 5 and S3). Furthermore, strains from vST1 to vST7 have a more similar distribution and number of copies of specific ISs compared to vST8 to vST12 (Fig. 5, Table S5); this is consistent with their phylogenetic similarities generated from CG analysis (Fig. 3). However, in some instances pINVs have undergone extended deletions affecting the localization and total number of particular ISs on the plasmid (Fig. 5, Table S5).

### Association of vST with serotypes

We then assessed whether the phylogenetic clades and vSTs were associated with specific serotypes, which have been frequently used to type *S. flexneri *. When not indicated, the serotype was predicted by identifying the serotype-specific O-antigen modification genes in the available genome sequences [25, 26]. It is interesting to note that strains from vST8 were all associated with serotype 6 (Table 1, Fig. 3b). This analysis confirms the high phylogenetic distance of serotype 6 (vST8) *S. flexneri* strains from other *S. flexneri* strains, consistent with the distinct evolutionary history proposed for this group [21, 23]. Strains carrying pINV plasmids of vST1 showed diverse serotypes while strains with plasmids belonging to vST11, vST12 and vST13 groups were all associated with serotype 3a_1_, and two out of three strains carrying vST7 pINV were serotype 1b (Tables 1 and S3). For the remaining vST groups, there was no obvious association with specific serotypes as they are only represented by a single strain (Table 1).

## Discussion

Several *Shigella* spp. infections have been recently reported in different western countries, most of which have been associated with travel and sexual activity, and have shown prevalence of *S. flexneri* and *Shigella sonnei* [37–44]. Schemes of MLST relying on the sequences of the seven housekeeping genes (*adk*, *fumC*, *gyrB*, *icd*, *mdh*, *purA* and *recA*) have been mostly used to type *Shigella *; however, they have limited resolution to distinguish strains [18]. Therefore, MLST has often been combined with serotyping which, nevertheless, has evident limitations especially for *S. flexneri* due to frequent serotype switching, as O antigen modification genes can be phage-encoded [17, 18]. It is interesting to note that most of available MLST schemes are based on chromosomal loci and ignore genes encoded on the virulence plasmid pINV [45–49]. However, sequence-based phylogenetic analyses have used both chromosomal and pINV genes to study the evolution and phylogenetic relationships of *Shigella* strains, and found that the pINVs of *Shigella* strains can be clustered in two major forms, pINV A and pINV B, and that this clustering is largely consistent with that based on chromosomal loci [21–23].

In this study, we used plasmid maintenance genes to study the molecular phylogeny of *S. flexneri* pINV, and the evolution and adaption of this plasmid to its host. In contrast to virulence genes, plasmid maintenance genes are well-conserved pINV elements that are generally unaffected by large deletions [15]. In particular, partitioning and TA systems are housekeeping elements of plasmids due to their role in plasmid persistence [11]. Following the analysis of 36 assembled pINVs and chromosomes, and 29 WGSs of *S. flexneri* strains from different geographical regions and belonging to different serotypes, we found that combining different alleles of the *vapBC* and *yacAB* TA systems*,* and the *parAB* partitioning system, allowed *S. flexneri* pINVs to be clustered into 13 distinct vSTs, and that this clustering was largely congruent with the phylogenetic analysis of the CGs. This suggests that despite the extensive plasticity of pINV, there are distinct regions of the plasmid that are not perturbed by IS-mediated rearrangements, and are remarkably conserved and have co-evolved with the chromosome. Furthermore, these results indicate that pINV, regardless of its plasticity, is a stable element, with no evidence that it is lost then re-acquired from different hosts by horizontal gene transfer. Phylogenetic analysis of genome sequences from *S. sonnei* clinical isolates found parallel relationship between chromosomal and plasmid lineages, indicating that the co-evolution of pINV with the chromosome may be a common feature of *Shigella* spp. [50].

From an evolutionary perspective, plasmids are considered as parasitic genetic elements that contribute to the evolution of their bacterial hosts by providing beneficial traits (e.g. virulence and antibiotic resistance) [51]. However, there is often a cost associated with plasmid carriage, which can result in antagonism between the plasmid and the chromosome [52]. Although changes on both the chromosome and the plasmid can alleviate fitness costs and lead to stable, long-term associations between bacteria and plasmids [51, 53–56], due to their mobile nature (i.e. conjugative plasmids) some plasmids, especially those conferring antibiotic resistance, are associated with multiple different bacterial hosts [57–59]. However, pINV is an unusual case due to a combination of specific features. pINV carries genes that are essential for the virulence of the bacterium (i.e. T3SS-related genes) and for its continued persistence inside a host bacterium [8, 9, 60, 61]. Furthermore, in contrast to many other large plasmids, pINV does not have an intact conjugation system, having only four transfer-associated genes [10], and it is still unclear if it is transferred between bacteria in nature. In the long term, these processes can result in the stable integration of plasmids into a chromosome; this event is thought to have given rise to the second chromosome of *Vibrio* [62–64]. Interestingly, phylogenetic analysis of the two *Vibrio* chromosomes has shown that despite significant sequence variation of some elements due to genetic mobility, the two chromosomes have co-evolved and their backbones share a common genetic history; indeed, the partitioning system locus *parA* of the second chromosome has been included in MLST for *Vibrionanceae* [62]. Similarly, in *Yersinia enterocolitica* and *Yersinia pseudotuberculosis *, the phylogenetic distances trees produced with chromosomal housekeeping genes resemble those derived from phylogenetic analysis of their virulence plasmids [65]. It would be interesting to assess long-lasting co-evolution of chromosome and plasmids in other pathogens that rely on virulence plasmids, including *Salmonella* and pathogenic *E. coli* [11].

In the era of genomics, WGS-based MLST (wgMLST) has become a valuable tool for epidemiological studies. However, specifically for *Shigella* subtyping, it has been recently suggested that a cgMLST approach is more robust than wgMLST, since, in contrast with the latter, the former eliminates genes associated with mobile genetic elements [66]. In particular, the EnteroBase cgMLST-based approach has been shown to have potential in routine surveillance and population structure analysis [67]. Similarly, studying pINV, we found that whole plasmid sequences are not informative for measuring phylogenetic distances between these plasmids because differences arising from spontaneous IS-mediated rearrangements do not reflect their evolutionary trajectories (data not shown). Instead, we demonstrated that three plasmid housekeeping loci, which are involved in plasmid maintenance, are sufficient to identify phylogenetic relationships between *S. flexneri* strains.

Interestingly, highly variable *Shigella* genetic elements such as ISs and serotypes do show a degree of correlation with the vST scheme. ISs are mobile genetic elements that contribute to the evolution and plasticity of the *Shigella* genome, by disrupting coding sequences and mediating inversions, deletions and translocations [10, 14, 15, 68]. Similar patterns of ISs are shared between pINV and the chromosome, indicating that there is a frequent inter-molecular exchange of ISs [68]. Of note, the distribution and numbers of ISs differ amongst *Shigella* spp., with distinct IS profiles being identified for each species [14]. In our work, we found that strains belonging to the same vST group possessed almost identical IS profiles on pINV, suggesting that IS transposition has a significant impact in the diversification of *S. flexneri* from other species as well as within a given species, and confirming it is still an active phenomenon. Furthermore, we provide a more detailed view of the diversification of plasmid IS profiles within the *S. flexneri* species; however, due to extended deletions in many pINV sequences and few representatives for some vSTs, results need to be confirmed by repeating the same analysis with more complete pINV sequences.

In contrast to ISs, the serotype was not always associated with vSTs. Association was only found for vST 8, 11, 12 and 13 with several serotypes found in vST1 strains; more intact and complete pINVs should be analysed to confirm this result, which is likely to result from the exchange of serotype-conferring phages. The high phylogenetic distance of the genome of serotype 6 strains (here classified as vST8) from other strains has been described previously and is reproduced by analysing only three plasmid maintenance loci [22, 23]. The LPS of serotype 6 has a distinct core structure and *rfb* gene cluster responsible for the biosynthesis of the O-antigen, which differs structurally from all other *S. flexneri* serotypes [69, 70]. This suggests that this serotype together with the genome underwent a very early diversification from other *S. flexneri* lineages; furthermore, due to differences in the LPS biosynthetic genes, it is not subject to the effect of serotype switching phages and plasmids.

In summary, this work shows the importance of pINV for tracing *S. flexneri* phylogeny and demonstrates how the conservation of plasmid maintenance genes, such those found on the *S. flexneri* pINV, can be exploited for highly discriminatory sequence typing as well as for the study of the phylogenetic relationship between the plasmid and its host chromosome. We believe this scheme could be expanded to other *Shigella* spp. and enteric pathogens that rely on virulence plasmids such as pathogenic *E. coli *, *Yersinia* and *Salmonella* [11]. However, the stability of the plasmids in the laboratory setting and the availability of assembled plasmid sequences are crucial for the development and effectiveness of such sequence type schemes.

## Supplementary Data

Supplementary material 1

Supplementary material 2

Supplementary material 3

Supplementary material 4

Supplementary material 5

Supplementary material 6
