# Supplementary material for: Virulence plasmid pINV as a genetic signature for Shigella flexneri phylogeny
Source: Microb Genom. 2022 Jun 27;8(6):mgen000846. doi: 10.1099/mgen.0.000846 (PMC9455713; doi:10.1099/mgen.0.000846)
Supplement: Supplementary material 1 [file mgen-8-846-s001.pdf]

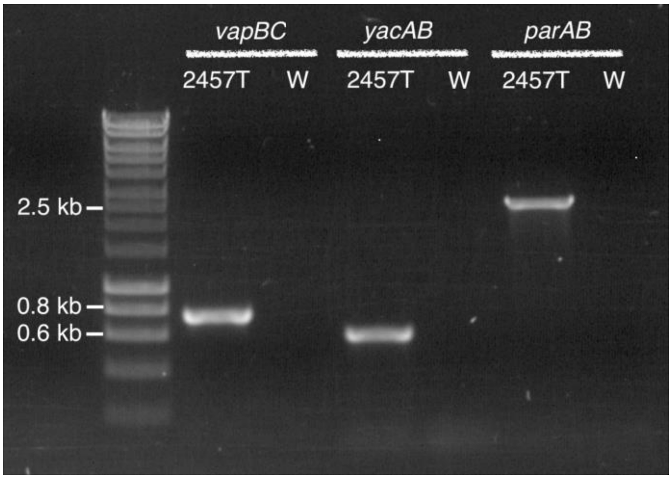

Supplementary Figure 1

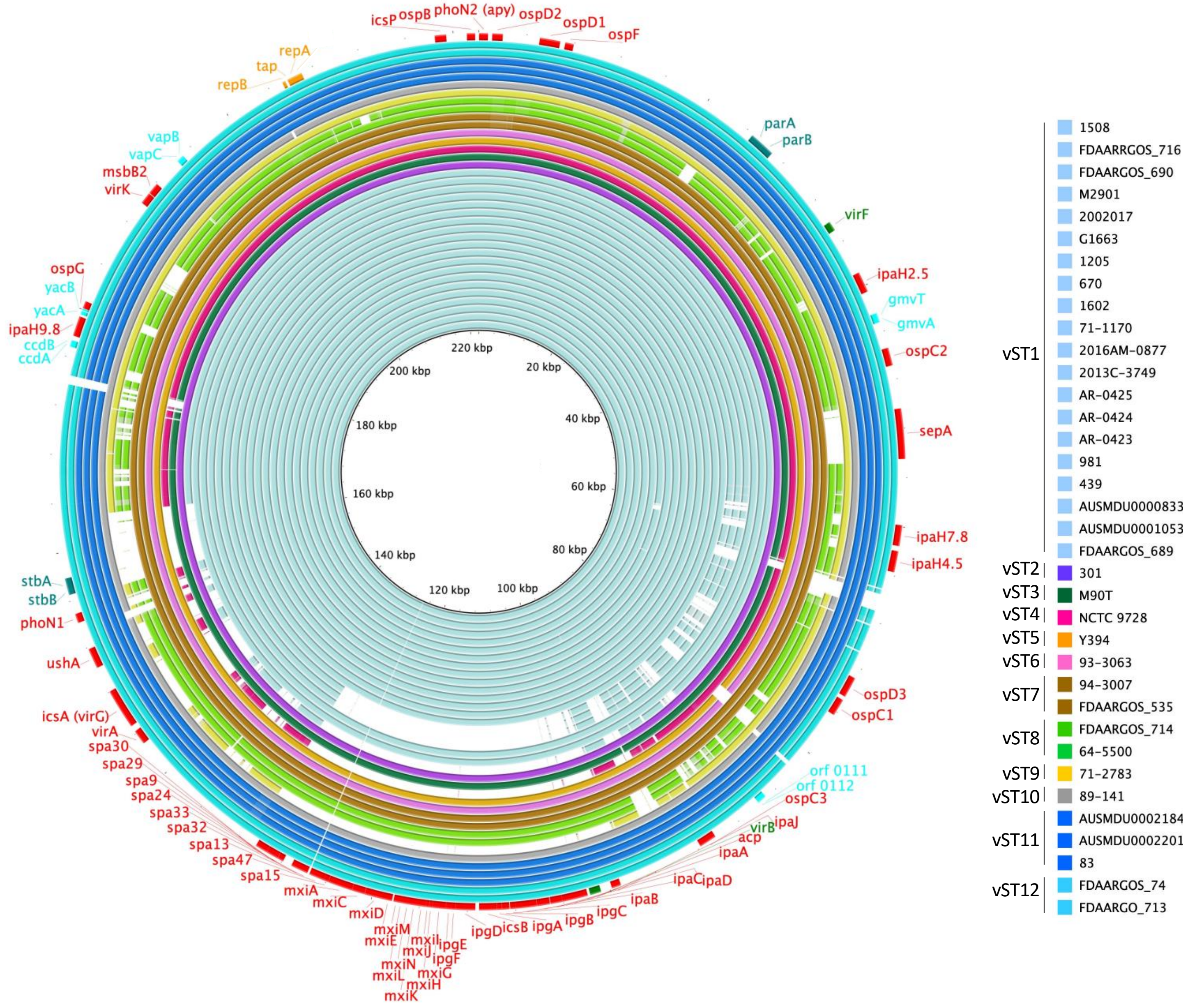

Supplementary Figure 2

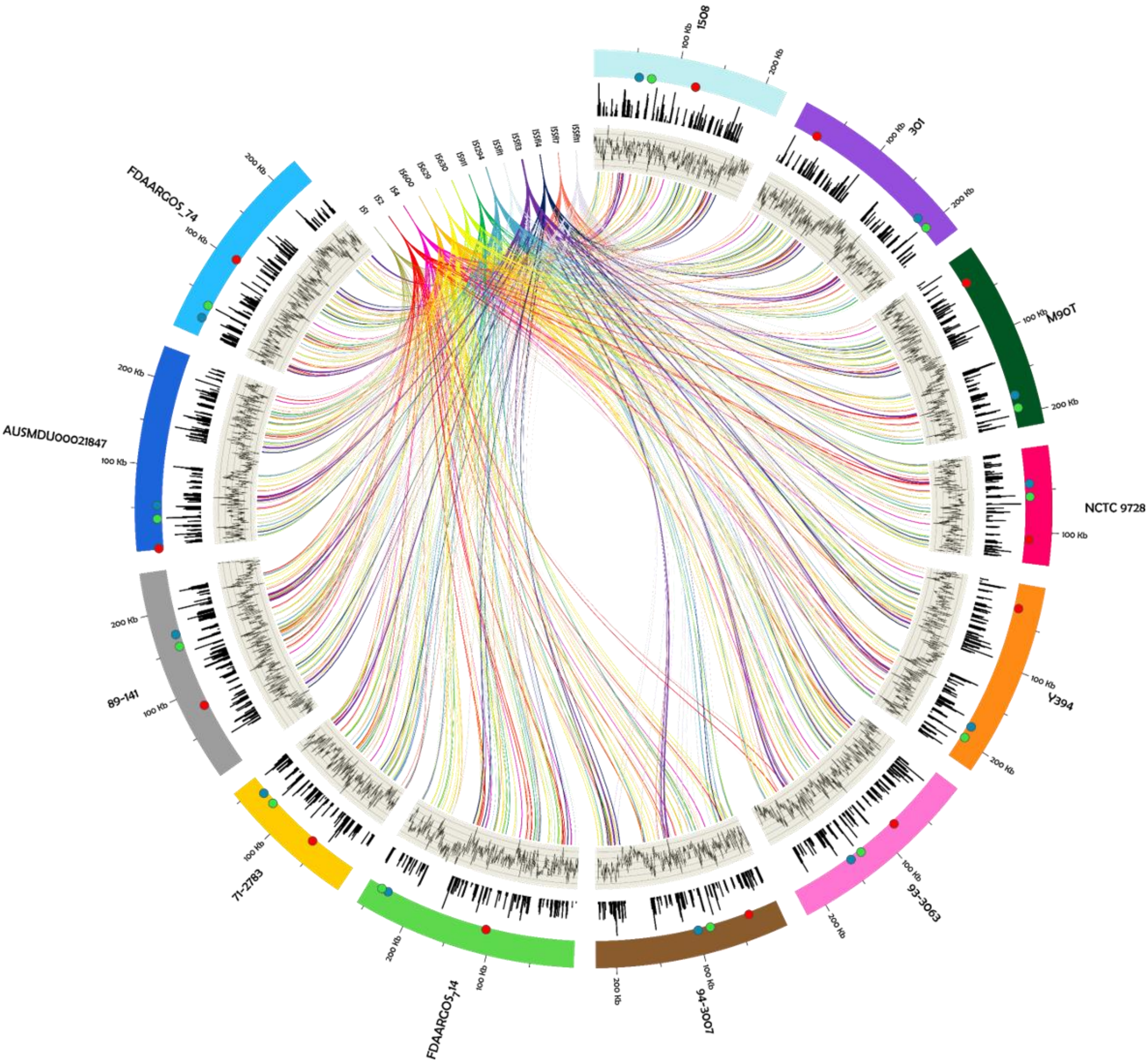

Supplementary Figure 3

Supplementary Table 1

Acc number Gen	Description NUCCORE	Length NUC	plasmidfinder	pmlst	*virK*	*virB*	*virF*	Analysis	*vapBC*	*ccdAB*	*gmvAT*	*orf 0111-2*	*yacAB*	*parAB*	*stbAB*
AF348706.1	Shigella flexneri 5a virulence plasmid pWR501, complete sequence	221851	IncFII_1, AY458016	IncF RST(F27:A-:B-)	pos	pos	pos	redundant, removed	100.00 %	100.00 %	100.00 %	100.00 %	100.00 %	100.00 %	100.00 %
AY879342.1	Shigella flexneri plasmid pSF5, complete sequence	136694	IncFII_1, AY458016	IncF RST(F27:A-:B-)	pos	neg	pos	removed vST3	100.00 %	100.00 %	100.00 %	100.00 %	100.00 %	100.00 %	-
NC_024996.1	Shigella flexneri 5a str. M90T plasmid pWR100, complete sequence	213494	IncFII_1, AY458016	IncF RST(F27:A-:B-)	pos	pos	pos	Studied	100.00 %	100.00 %	100.00 %	100.00 %	100.00 %	100.00 %	100.00 %
CP026800.1	Shigella flexneri strain NCTC 9728 plasmid unnamed, complete sequence	135368	IncFII_1, AY458016	IncF RST(F27:A-:B-)	pos	neg	pos	Studied	100.00 %	100.00 %	100.00 %	100.00 %	100.00 %	99.95 %	-
AF386526.1	Shigella flexneri 2a str. 301 plasmid pCP301, complete sequence	221618	IncFII_1, AY458016	IncF RST(F27:A-:B-)	pos	pos	pos	Studied	99.84 %	100.00 %	100.00 %	99.74 %	99.81 %	99.82 %	99.85 %
CP030773.1	Shigella flexneri 1c strain Y394 plasmid pINV-Y394, complete sequence	221293	IncFII_1, AY458016	IncF RST(F27:A-:B-)	pos	pos	pos	Studied	100.00 %	100.00 %	100.00 %	99.74 %	99.81 %	99.82 %	99.85 %
CP030916.1	Shigella flexneri 2a strain 1508 plasmid pSF150801	222126	IncFII_1, AY458016	IncF RST(F27:A-:B-)	pos	pos	pos	Studied	99.84 %	100.00 %	100.00 %	99.87 %	99.81 %	99.86 %	99.78 %
CP050984.1	Shigella flexneri strain FDAARGOS_716 plasmid unnamed1, complete sequence	231172	IncFII_1, AY458016	IncF RST(F27:A-:B-)	pos	pos	pos	Studied	99.84 %	100.00 %	100.00 %	99.87 %	99.81 %	99.86 %	99.78 %
CP055137.1	Shigella flexneri strain FDAARGOS_690 plasmid unnamed1, complete sequence	228903	IncFII_1, AY458016	IncF RST(F27:A-:B-)	pos	pos	pos	Studied	99.84 %	100.00 %	100.00 %	99.87 %	99.81 %	99.86 %	99.85 %
CP058591.1	Shigella flexneri strain M2901 plasmid unnamed2, complete sequence	224303	IncFII_1, AY458016	IncF RST(F27:A-:B-)	pos	pos	pos	Studied	99.84 %	100.00 %	100.00 %	99.87 %	99.81 %	99.86 %	99.78 %
CP001384.1	Shigella flexneri 2002017 plasmid pSFxv_1, complete sequence	223364	IncFII_1, AY458016	IncF RST(F27:A-:B-)	pos	pos	pos	Studied	99.84 %	100.00 %	100.00 %	99.87 %	99.81 %	99.86 %	99.85 %
CP007038.1	Shigella flexneri G1663 plasmid pG1663, complete sequence	222586	IncFII_1, AY458016	IncF RST(F27:A-:B-)	pos	pos	pos	Studied	99.84 %	100.00 %	100.00 %	99.87 %	99.81 %	99.86 %	99.85 %
CP012142.1	Shigella flexneri 4c strain 1205 plasmid 1205p2, complete sequence	222060	IncFII_1, AY458016	IncF RST(F27:A-:B-)	pos	pos	pos	Studied	99.84 %	100.00 %	100.00 %	99.87 %	99.81 %	99.86 %	99.85 %
CP020087.1	Shigella flexneri 1a strain 0670 plasmid unnamed1, complete sequence	228834	IncFII_1, AY458016	IncF RST(F27:A-:B-)	pos	pos	pos	Studied	99.84 %	100.00 %	100.00 %	99.87 %	99.81 %	99.86 %	99.85 %
CP020337.1	Shigella flexneri 4c strain 1602 plasmid unnamed1, complete sequence	223354	IncFII_1, AY458016	IncF RST(F27:A-:B-)	pos	pos	pos	Studied	99.84 %	100.00 %	100.00 %	99.87 %	99.81 %	99.86 %	99.85 %
CP024476.1	Shigella flexneri 7b strain 94-3007 plasmid unnamed3, complete sequence	220282	IncFII_1, AY458016	IncF RST(F27:A-:B-)	pos	pos	pos	Studied	99.84 %	100.00 %	100.00 %	99.87 %	99.81 %	99.86 %	99.78 %
CP026771.1	Shigella flexneri Y strain 93-3063 plasmid unnamed3, complete sequence	220759	IncFII_1, AY458016	IncF RST(F27:A-:B-)	pos	pos	pos	Studied	100.00 %	100.00 %	100.00 %	99.87 %	99.81 %	99.86 %	99.85 %
CP026794.1	Shigella flexneri strain 74-1170 plasmid unnamed	251323	IncFII_1, AY458016	IncF RST(F27:A-:B-)	pos	pos	pos	Studied	99.84 %	100.00 %	100.00 %	99.87 %	99.81 %	99.86 %	99.85 %
CP033511.1	Shigella flexneri strain 2016AM-0877 plasmid p2016AM-0877, complete sequence	222142	IncFII_1, AY458016	IncF RST(F27:A-:B-)	pos	pos	pos	Studied	99.84 %	100.00 %	100.00 %	99.87 %	99.81 %	99.86 %	99.85 %
CP034059.1	Shigella flexneri strain FDAARGOS_535 plasmid unnamed1, complete sequence	217451	IncFII_1, AY458016	IncF RST(F27:A-:B-)	pos	pos	pos	Studied	99.84 %	100.00 %	100.00 %	99.87 %	99.81 %	99.86 %	99.78 %
CP034933.1	Shigella flexneri strain 2013C-3749 plasmid p2013C-3749-2, complete sequence	223405	IncFII_1, AY458016	IncF RST(F27:A-:B-)	pos	pos	pos	Studied	99.84 %	100.00 %	100.00 %	99.87 %	99.81 %	99.86 %	99.85 %
CP044154.1	Shigella flexneri strain AR-0425 plasmid pAR-0425-2, complete sequence	222114	IncFII_1, AY458016	IncF RST(F27:A-:B-)	pos	pos	pos	Studied	99.84 %	100.00 %	100.00 %	99.87 %	99.81 %	99.86 %	99.85 %
CP044157.1	Shigella flexneri strain AR-0424 plasmid pAR-0424-2, complete sequence	226601	IncFII_1, AY458016	IncF RST(F27:A-:B-)	pos	pos	pos	Studied	99.84 %	100.00 %	100.00 %	99.87 %	99.81 %	99.86 %	99.85 %
CP044160.1	Shigella flexneri strain AR-0423 plasmid pAR-0423-2, complete sequence	146462	IncFII_1, AY458016	IncF RST(F27:A-:B-)	pos	neg	pos	Studied	99.84 %	100.00 %	100.00 %	-	99.81 %	99.86 %	99.85 %
CP012138.1	Shigella flexneri 2a strain 981 plasmid 981p1, complete sequence	144188	IncFII_1, AY458016	IncF RST(F27:A-:B-)	pos	neg	pos	Studied	99.84 %	100.00 %	100.00 %	-	99.81 %	99.86 %	99.85 %
CP020343.1	Shigella flexneri 1a strain 0439 plasmid unnamed1, complete sequence	144194	IncFII_1, AY458016	IncF RST(F27:A-:B-)	pos	neg	pos	Studied	99.84 %	100.00 %	100.00 %	-	99.81 %	99.86 %	99.85 %
CP026791.1	Shigella flexneri strain 61-4982 plasmid unnamed, complete sequence	59834	IncFII_1, AY458016	IncF RST(F27:A-:B-) - imperfect	pos	neg	neg	not studied	100.00 %	100.00 %	100.00 %	-	99.81 %	-	-
CP026812.1	Shigella flexneri strain 64-5500 plasmid unnamed, complete sequence	181479	IncFII_1, AY458016	IncF RST(F27:A-:B-)	pos	pos	pos	Studied	99.52 %	100.00 %	99.74 %	99.87 %	100.00 %	99.86 %	-
LR213456.1	Shigella flexneri strain AUSMDU00008332 isolate AUSMDU00008332 plasmid 2	234171	IncFII_1, AY458016	IncF RST(F27:A-:B-)	pos	pos	pos	Studied	99.84 %	100.00 %	99.87 %	99.87 %	99.81 %	99.86 %	99.78 %
CP045942.1	Shigella flexneri 2a strain AUSMDU00010535 plasmid pAUSMDU00010535_01, complete sequence	234182	IncFII_1, AY458016	IncF RST(F27:A-:B-)	pos	pos	pos	Studied	99.84 %	100.00 %	99.87 %	99.87 %	99.81 %	99.86 %	99.78 %
CP055125.1	Shigella flexneri strain FDAARGOS_714 plasmid unnamed1	255031	IncFII_1, AY458016, IncFII_1, AY	IncF RST(F27:F27:A-:B-)	pos	pos	pos	Studied	99.52 %	100.00 %	99.61 %	99.87 %	100.00 %	99.86 %	-
CP024472.1	Shigella flexneri strain 71-2783 plasmid unnamed2, complete sequence	159299	IncFII_1, AY458016	IncF RST(F27:A-:B-)	pos	neg	pos	Studied	99.68 %	98.29 %	99.74 %	99.87 %	100.00 %	99.91 %	-
CP026804.1	Shigella flexneri strain 89-141 plasmid unnamed1	245004	IncFII_1, AY458016	IncF RST(F27:A-:B-)	pos	pos	pos	Studied	99.84 %	99.81 %	99.74 %	99.87 %	100.00 %	99.91 %	99.85 %
LR861786.1	Shigella flexneri strain AUSMDU00021847 genome assembly, plasmid: 2	233332	IncFII_1, AY458016	IncF RST(F27:A-:B-)	pos	pos	pos	Studied	100.00 %	99.81 %	99.87 %	99.74 %	100.00 %	99.91 %	99.85 %
LR861789.1	Shigella flexneri strain AUSMDU00022017 genome assembly, plasmid: 2	233274	IncFII_1, AY458016	IncF RST(F27:A-:B-)	pos	neg	pos	Studied	100.00 %	99.81 %	99.87 %	99.74 %	100.00 %	99.91 %	99.85 %
LR878366.1	Shigella flexneri isolate 83 genome assembly, plasmid: 83_VP	231165	IncFII_1, AY458016	IncF RST(F27:A-:B-)	pos	pos	pos	Studied	100.00 %	99.81 %	99.87 %	99.74 %	100.00 %	99.91 %	99.85 %
CP054891.1	Shigella flexneri strain FDAARGOS_713 plasmid unnamed1, complete sequence	220335	IncFII_1, AY458016	IncF RST(F27:A-:B-)	pos	pos	pos	Studied	100.00 %	99.81 %	99.87 %	99.74 %	99.81 %	99.91 %	99.85 %
CP026099.1	Shigella flexneri strain FDAARGOS_74 plasmid unnamed	229413	IncFII_1, AY458016	IncF RST(F27:A-:B-)	pos	pos	pos	Studied	100.00 %	99.81 %	99.87 %	99.74 %	99.81 %	99.91 %	99.85 %
CP026789.1	Shigella flexneri 2a strain ATCC 29903 plasmid unnamed1, complete sequence	113130	IncFII_1, AY458016	IncF RST(F27:A-:B-)	neg	pos	pos	not studied	99.84 %	-	100.00 %	-	-	99.86 %	-
CP054887.1	Shigella flexneri strain FDAARGOS_689 plasmid unnamed1	176253	IncFII_1, AY458016	IncF RST(F27:A-:B-)	pos	neg	pos	Studied	99.84 %	100.00 %	100.00 %	-	99.81 %	99.86 %	-
CP054888.1	Shigella flexneri strain FDAARGOS_689 plasmid unnamed2	51857	No hit-Untypable					untypable							
CP041619.1	Shigella flexneri strain C32 plasmid pC32_1, complete sequence	90682	IncFIB(AP001918)_1, AP001918	IncF RST(F-:A1:B10)				No virulence plasmid							
CP024474.1	Shigella flexneri 7b strain 94-3007 plasmid unnamed1, complete sequence	69554	IncFIB(K)_1, JN233704	not available				No virulence plasmid							
LR213454.1	Shigella flexneri strain AUSMDU00008355 isolate AUSMDU00008355 plasmid 3	69446	IncFIB(K)_1, JN233704	not available				No virulence plasmid							
CP020344.1	Shigella flexneri 1a strain 0439 plasmid unnamed2, complete sequence	78308	IncFIB(K)_1, JN233704, IncN_1_	not available				No virulence plasmid							
CP026764.1	Shigella flexneri strain 04-3145 plasmid unnamed, complete sequence	68319	IncFIB(K)_1, JN233704, IncN_1	not available				No virulence plasmid							
KY848295.1	Shigella flexneri Y strain RC960 plasmid pRC960-1, complete sequence	75231	IncFIB(K)_1, JN233704, IncN_1_	not available				No virulence plasmid							
CP020338.1	Shigella flexneri 4c strain 1602 plasmid unnamed2, complete sequence	95633	IncFII(pHN7A8)_1, JN232517	IncF RST(F103:A-:B-)				No virulence plasmid							
CP024475.1	Shigella flexneri 7b strain 94-3007 plasmid unnamed2, complete sequence	82833	IncFII(pSE11)_1, AP009242	IncF RST(F35:A-:B-)				No virulence plasmid							
CP044156.1	Shigella flexneri strain AR-0424 plasmid pAR-0424-1, complete sequence	79831	IncFII(pSE11)_1, AP009242	IncF RST(F35:A-:B-)				No virulence plasmid							
CP045943.1	Shigella flexneri 2a strain AUSMDU00010535 plasmid pAUSMDU00010535_02, complete sequence	83548	IncFII(pSE11)_1, AP009242	IncF RST(F35:A-:B-)				No virulence plasmid							
LR213453.1	Shigella flexneri strain AUSMDU00008355 isolate AUSMDU00008355 plasmid 2	82887	IncFII(pSE11)_1, AP009242	IncF RST(F35:A-:B-)				No virulence plasmid							
LR213457.1	Shigella flexneri strain AUSMDU00008332 isolate AUSMDU00008332 plasmid 3	76615	IncFII(pSE11)_1, AP009242	IncF RST(F35:A-:B-)				No virulence plasmid							
AP000342.1	Shigella flexneri 2b plasmid R100 DNA, complete sequence	94281	IncFII_1, AY458016	IncF RST(F2:A-:B-)				No virulence plasmid							
CP012139.1	Shigella flexneri 2a strain 981 plasmid 981p2, complete sequence	86407	IncFII_1, AY458016	IncF RST(F2:A-:B-)				No virulence plasmid							
CP020340.1	Shigella flexneri 4c strain 0702 plasmid unnamed1, complete sequence	73096	IncFII_1, AY458016	IncF RST(F2:A-:B-)				No virulence plasmid							
CP034932.1	Shigella flexneri strain 2013C-3749 plasmid p2013C-3749-1, complete sequence	71053	IncFII_1, AY458016	IncF RST(F2:A-:B-)				No virulence plasmid							
KJ201887.1	Shigella flexneri 4c strain 072 plasmid pSF07201, complete sequence	75335	IncFII_1, AY458016	IncF RST(F2:A-:B-)				No virulence plasmid							
HE578057.1	Shigella sp. MO17 plasmid pMO17_54, complete sequence	54291	IncX1_4, JN935898	not available				No virulence plasmid							
CP045523.1	Shigella flexneri strain 5908.2 plasmid p5908-2	137368	IncI1-I(Gamma)_1, AP005147; Ir	IncI1 ST31				No virulence plasmid							
CP026790.1	Shigella flexneri 2a strain ATCC 29903 plasmid unnamed2, complete sequence	165702	IncHI1A_1, AF250878|IncHI1B(F	not available				No virulence plasmid							
CP012141.1	Shigella flexneri 4c strain 1205 plasmid 1205p1, complete sequence	248368	IncHI2A_1, BX664015|IncHI2_1	IncHI2 ST3				No virulence plasmid							
CP041621.1	Shigella flexneri strain C32 plasmid pC32_2, complete sequence	88076	IncI1-I(Gamma)_1, AP005147	not available				No virulence plasmid							
CP034061.1	Shigella flexneri strain FDAARGOS_535 plasmid unnamed2	57818	IncI2(Delta)_1, AP002527	not available				No virulence plasmid							
CP012143.1	Shigella flexneri 4c strain 1205 plasmid 1205p3, complete sequence	42002	IncN_1, AY046276	IncN ST1				No virulence plasmid							
CP044159.1	Shigella flexneri strain AR-0423 plasmid pAR-0423-1, complete sequence	59694	IncN_1, AY046276|ColRNAI_1, I	IncN ST5				No virulence plasmid							
CP030774.1	Shigella flexneri 1c strain Y394 plasmid pNV-Y394, complete sequence	10866	IncQ1_1, M28829	not available				No virulence plasmid							
HE578058.1	Shigella sp. LN126 plasmid pLN126_33, complete sequence	32533	IncX1_1, EU370913	not available				No virulence plasmid							
CP020088.1	Shigella flexneri 1a strain 0670 plasmid unnamed2, complete sequence	47674	IncX1_1, EU370913	not available				No virulence plasmid							
CP020341.1	Shigella flexneri 4c strain 0702 plasmid unnamed2, complete sequence	47688	IncX1_1, EU370913	not available				No virulence plasmid							
CP044153.1	Shigella flexneri strain AR-0425 plasmid pAR-0425-1, complete sequence	27094	IncX1_1, EU370913	not available				No virulence plasmid							
KJ201886.1	Shigella flexneri 4c strain 072 plasmid pSF07202, complete sequence	47669	IncX1_1, EU370913	not available				No virulence plasmid							
CP012734.1	Shigella flexneri 1a strain 0228 plasmid unnamed, complete sequence	63412	IncX1_1, EU370913	not available				No virulence plasmid							
CP026770.1	Shigella flexneri Y strain 93-3063 plasmid unnamed2	71925	IncB/O/K/Z_2, GU256641	not available				No virulence plasmid							
CP054894.1	Shigella flexneri strain FDAARGOS_713 plasmid unnamed3, complete sequence	2087	Col(BS512)_1, NC_010656	not available				No virulence plasmid							
JF813186.1	Shigella flexneri plasmid pSF301-1, complete sequence	1549	Col(MG828)_1, NC_008486	not available				No virulence plasmid							
CP034062.1	Shigella flexneri strain FDAARGOS_535 plasmid unnamed3, complete sequence	1538	Col(MG828)_1, NC_008486	not available				No virulence plasmid							
CP001385	Shigella flexneri 2002017 plasmid pSFxv_2, complete sequence	6850	Col(pHAD28)_1, KU674895	not available				No virulence plasmid							
CP001386.1	Shigella flexneri 2002017 plasmid pSFxv_3, complete sequence	6200	Col(pHAD28)_1, KU674895	not available				No virulence plasmid							
KC020049.1	Shigella flexneri strain HN006 plasmid pSFyv, complete sequence	6852	Col(pHAD28)_1, KU674895	not available				No virulence plasmid							
CP045944.1	Shigella flexneri 2a strain AUSMDU00010535 plasmid pAUSMDU00010535_03, complete sequence	4692	Col156_1, NC_009781	not available				No virulence plasmid							
CP054893.1	Shigella flexneri strain FDAARGOS_713 plasmid unnamed2, complete sequence	2442	Col3_SH_CP054893.1	not available				No virulence plasmid							
CP054889	Shigella flexneri strain FDAARGOS_689 plasmid unnamed3	4042	ColRNAI_1, DQ298019	not available				No virulence plasmid							
CP054914.1	Shigella flexneri strain FDAARGOS_691 plasmid unnamed1, complete sequence	4042	ColRNAI_1, DQ298019	not available				No virulence plasmid							
CP042979.1	Shigella flexneri Y strain PE577 plasmid unnamed1, complete sequence	3990	ColRNAI_1, DQ298019	not available				No virulence plasmid							
CP054890	Shigella flexneri strain FDAARGOS_689 plasmid unnamed4	3180	ColRNAI_2_SH_CP054915.1	not available				No virulence plasmid							
CP054895.1	Shigella flexneri strain FDAARGOS_713 plasmid unnamed3, complete sequence	2664	ColRNAI_2_SH_CP054915.1	not available				No virulence plasmid							
CP054915.1	Shigella flexneri strain FDAARGOS_691 plasmid unnamed2, complete sequence	3180	ColRNAI_2_SH_CP054915.1	not available				No virulence plasmid							
AY028316.1	Shigella flexneri 2a plasmid p2457TS2, complete sequence	3179	ColRNAI_2_SH_CP054915.1	not available				No virulence plasmid							
CP001387.1	Shigella flexneri 2002017 plasmid pSFxv_4, complete sequence	4042	ColRNAI_2_SH_CP054915.1	not available				No virulence plasmid							
CP001388.1	Shigella flexneri 2002017 plasmid pSFxv_5, complete sequence	3180	ColRNAI_2_SH_CP054915.1	not available				No virulence plasmid							
JF813187.1	Shigella flexneri plasmid pSF301-2, complete sequence	3178	ColRNAI_2_SH_CP054915.1	not available				No virulence plasmid							
JF813188.1	Shigella flexneri strain 2a 301 plasmid pSF301-3, complete sequence	4043	ColRNAI_2_SH_CP054915.1	not available				No virulence plasmid							
CP034063.1	Shigella flexneri strain FDAARGOS_535 plasmid unnamed4	3920	ColRNAI_2_SH_CP054915.1	not available				No virulence plasmid							
CP034064.1	Shigella flexneri strain FDAARGOS_535 plasmid unnamed5	3057	ColRNAI_2_SH_CP054915.1	not available				No virulence plasmid							
CP034065.1	Shigella flexneri strain FDAARGOS_535 plasmid unnamed6	2551	ColRNAI_2_SH_CP054915.1	not available				No virulence plasmid							
CP042978.1	Shigella flexneri Y strain PE577 plasmid unnamed2, complete sequence	3128	ColRNAI_2_SH_CP054915.1	not available				No virulence plasmid							
CP012732.1	Shigella flexneri 1a strain 0228 plasmid unnamed, complete sequence	25194	phage	not available				No virulence plasmid							
CP012733.1	Shigella flexneri 1a strain 0228 plasmid unnamed, complete sequence	41686	phage replication protein O	not available				No virulence plasmid							
CP012736.1	Shigella flexneri 1a strain 0228 plasmid unnamed, complete sequence	24419	phage replication protein O	not available				No virulence plasmid							

Supplementary Table 2

IS	Length (bp)	Identity (%)
**IS*1294***	1688	99
**IS*600***	1244-1264	98
**IS*629***	1305-1310	97
**IS*630***	1153	98
**IS*1***	768	98
**IS*2***	1331	99
**IS*4***	1426	99
**IS*911***	1250	99
**IS*Sfl1***	1301	99
**IS*Sfl3***	2729	98
**IS*Sfl4***	1451	99
**IS*Sfl7***	916	99
**IS*Sfl11***	1139	99

Supplementary Table 3

WGS project/ Accession	Strain	Serotype	Country of origin	*vapBC* allele	*parAB* allele	*yacAB* allele	vST group
AATWQA	PNUSAE049295	Y_2	USA	1	1	1	**1**
AATWQE	892751	$2a_2$	UK				
AATWQF	892754	$2a_2$	UK				
AATWQG	892729	$2a_2$	UK				
AATWQH	892701	$2a_2$	UK				
AATWQO	892741	$3a_1$	UK				
AAZDPL	PNUSAE024364	Y_2	USA				
AAZDPO	PNUSAE024363	Y_2	USA				
AAZJOY	PNUSAE012744	$2a_2$	USA				
PRJNA408	2457T	$2a_2$	USA				
AATWQI	PNUSAE049227	1b	USA	6	1	1	**7**
AATWQC	892752	$3a_1$	UK	2	5	2	**11**
AATWQD	892705	$3a_1$	UK				
AATWQL	PNUSAE049225	$3a_1$	USA				
ATTWQP	PNUSAE049193	$3a_1$	USA				
AAZDPI	PNUSAE067434	$3a_1$	USA				
AAZJOB	PNUSAE021406	$3a_1$	USA				
AAZJOG	AUSMDU00007269	$3a_1$	Australia				
AAZJOM	671916	$3a_1$?	UK				
AAZJON	PNUSAE020468	$3a_1$	USA				
AAZJOQ	PNUSAE020469	$3a_1$	USA				
AAZJOT	PNUSAE018503	$3a_1$	USA				
AAZJOV	PNUSAE015436	$3a_1$	USA				
AAZJOW	PNUSAE012914	$3a_1$	USA				
AAZJOZ	E7311	$3a_1$	USA				
AAZJOI	AUSMDU00010031	$3a_1$	Australia	2	5	3	**12**
AAZJOJ	673667	$3a_1$	UK				
AAZJOK	AUSMDU00013122	$3a_1$	Australia				
AAZJOO	664960	$3a_1$	UK				
AAZJOL	PNUSAE021273	$3a_1$	USA	2	8	2	**13**

Supplementary Table 4

Isolate	vST	ST	HC0	HC2	HC5	HC10	HC20	HC50	HC100	HC200	HC400	HC1100	HC1500	HC2000	HC2350
AUSMDU00010535	1	137959	122843	112695	293	293	293	293	206	192	192	192	192	192	1
G1663	1	1473	1473	1473	1473	1473	1473	1473	1473	192	192	192	192	192	1
M2901	1	126173	126173	126173	126173	92262	92262	92262	192	192	192	192	192	192	1
0439	1	79138	79138	79138	79138	79138	79138	195	192	192	192	192	192	192	1
1205	1	1479	1479	1479	1479	1479	1479	1479	192	192	192	192	192	192	1
1508	1	137408	137408	137408	79480	79480	79480	79480	192	192	192	192	192	192	1
1602	1	79135	79135	79135	79135	79135	79135	79135	192	192	192	192	192	192	1
2002017	1	1474	1474	1474	1474	1474	1474	1474	192	192	192	192	192	192	1
2016AM-0877	1	80075	79513	37410	37410	37410	37410	196	192	192	192	192	192	192	1
2457T	1	1464	1464	1464	1464	1464	1464	1464	1464	192	192	192	192	192	1
74-1170	1	80183	79300	79300	79300	17234	17234	17234	17234	192	192	192	192	192	1
0670	1	79139	79139	79139	79139	79139	79139	79139	192	192	192	192	192	192	1
981	1	1482	1482	1482	1482	1482	1482	1482	192	192	192	192	192	192	1
AUSMDU00008332	1	85712	85712	85712	293	293	293	293	206	192	192	192	192	192	1
FDAARGOS_689	1	98537	98537	98537	98537	98537	98537	206	206	192	192	192	192	192	1
FDAARGOS_716	1	98257	98257	98257	98257	98257	98257	1517	1517	192	192	192	192	192	1
892751	1	132296	132296	132296	132296	132296	132296	196	192	192	192	192	192	192	1
892754	1	132285	132285	132285	132285	34745	34745	34745	206	192	192	192	192	192	1
892729	1	132288	132288	83474	83474	83474	28430	196	192	192	192	192	192	192	1
892701	1	132290	132290	126255	126255	126255	126255	196	192	192	192	192	192	192	1
892741	1	132215	132215	66363	47221	34759	1549	1511	544	544	544	204	192	192	1
89-141	10	80185	79256	79256	79256	79256	79256	79256	11776	11594	11594	11594	192	192	1
892752	11	132286	80790	66363	47221	34759	1549	1511	544	544	544	204	192	192	1
892705	11	132287	131888	94184	47221	34759	1549	1511	544	544	544	204	192	192	1
AUSMDU00007269	11	120426	120426	120426	120426	120426	62300	36891	36891	30456	544	204	192	192	1
671916	11	83384	83384	83384	83384	83384	83384	63979	63979	30456	544	204	192	192	1
PNUSAE012914	11	62300	62300	62300	62300	62300	62300	36891	36891	30456	544	204	192	192	1
FDAARGOS_713	12	98264	98264	98264	98264	98264	98264	11959	11959	11959	544	204	192	192	1
FDAARGOS_74	12	137400	137400	17137	17137	17137	17137	11959	11959	11959	544	204	192	192	1
AUSMDU00010031	12	120078	120078	120078	120078	120078	120078	11959	11959	11959	544	204	192	192	1
673667	12	84438	84438	84438	84438	84438	84438	11959	11959	11959	544	204	192	192	1
AUSMDU00013122	12	120281	120281	120281	64012	64012	12030	11959	11959	11959	544	204	192	192	1
664960	12	91846	91846	83004	83004	83004	83004	13943	11959	11959	544	204	192	192	1
301	2	1472	1472	1472	1472	1472	1472	1472	1472	192	192	192	192	192	1
M90T	3	80285	78983	13517	13517	13517	13517	13517	13517	11337	1468	1468	192	192	1
NCTC 9728	4	80190	79312	79312	79312	11335	11335	11335	11335	11335	1468	1468	192	192	1
Y394	5	137399	137399	137399	79169	79169	79169	11802	327	327	327	192	192	192	1
93-3063	6	79267	79267	79267	79267	79267	79267	79267	79267	17232	327	192	192	192	1
94-3007	7	79183	79183	79183	79183	79183	680	680	433	237	237	192	192	192	1
FDAARGOS_535	7	137407	137407	137407	79524	79524	79524	30679	433	237	237	192	192	192	1
64-5500	8	80201	79264	79264	79264	79264	79264	79264	79264	11126	11126	1465	1465	1465	1
FDAARGOS_714	8	98265	98265	98265	98265	98265	98265	22885	22885	11126	11126	1465	1465	1465	1
71-2783	9	80215	79193	79193	17194	17194	17194	14123	14123	14123	543	543	192	192	1
61-4982	NT	80196	79293	79293	79293	79293	79293	17200	17200	14126	327	192	192	192	1
ATCC_29903	NT	79294	79294	79294	79294	4908	4908	1464	1464	192	192	192	192	192	1
2013C-3749	1	ND	ND	ND	ND	ND	ND	ND	ND	ND	ND	ND	ND	ND	ND
AR-0423	1	ND	ND	ND	ND	ND	ND	ND	ND	ND	ND	ND	ND	ND	ND
AR-0424	1	ND	ND	ND	ND	ND	ND	ND	ND	ND	ND	ND	ND	ND	ND
AR-0425	1	ND	ND	ND	ND	ND	ND	ND	ND	ND	ND	ND	ND	ND	ND
FDAARGOS_690	1	ND	ND	ND	ND	ND	ND	ND	ND	ND	ND	ND	ND	ND	ND
PNUSAE024363	1	ND	ND	ND	ND	ND	ND	ND	ND	ND	ND	ND	ND	ND	ND
PNUSAE024364	1	ND	ND	ND	ND	ND	ND	ND	ND	ND	ND	ND	ND	ND	ND
PNUSAE012744	1	ND	ND	ND	ND	ND	ND	ND	ND	ND	ND	ND	ND	ND	ND
PNUSAE049295	1	ND	ND	ND	ND	ND	ND	ND	ND	ND	ND	ND	ND	ND	ND
83	11	ND	ND	ND	ND	ND	ND	ND	ND	ND	ND	ND	ND	ND	ND
AUSMDU00021847	11	ND	ND	ND	ND	ND	ND	ND	ND	ND	ND	ND	ND	ND	ND
AUSMDU00022017	11	ND	ND	ND	ND	ND	ND	ND	ND	ND	ND	ND	ND	ND	ND
PNUSAE049225	11	ND	ND	ND	ND	ND	ND	ND	ND	ND	ND	ND	ND	ND	ND
PNUSAE049193	11	ND	ND	ND	ND	ND	ND	ND	ND	ND	ND	ND	ND	ND	ND
PNUSAE067434	11	ND	ND	ND	ND	ND	ND	ND	ND	ND	ND	ND	ND	ND	ND
PNUSAE021406	11	ND	ND	ND	ND	ND	ND	ND	ND	ND	ND	ND	ND	ND	ND
PNUSAE020468	11	ND	ND	ND	ND	ND	ND	ND	ND	ND	ND	ND	ND	ND	ND
PNUSAE020469	11	ND	ND	ND	ND	ND	ND	ND	ND	ND	ND	ND	ND	ND	ND
PNUSAE018503	11	ND	ND	ND	ND	ND	ND	ND	ND	ND	ND	ND	ND	ND	ND
PNUSAE015436	11	ND	ND	ND	ND	ND	ND	ND	ND	ND	ND	ND	ND	ND	ND
E7311	11	ND	ND	ND	ND	ND	ND	ND	ND	ND	ND	ND	ND	ND	ND
PNUSAE021273	13	ND	ND	ND	ND	ND	ND	ND	ND	ND	ND	ND	ND	ND	ND
PNUSAE049227	7	ND	ND	ND	ND	ND	ND	ND	ND	ND	ND	ND	ND	ND	ND

Supplementary Table 5

Strain	***IS1294***	***IS600***	***IS629***	***IS630***	***IS1***	***IS2***	***IS4***	***IS911***	***ISSfl1***	***ISSfl3***	***ISSfl4***	***ISSfl7***	***ISSfl11***
1508	5	5	8	1	2	2	1	**0**	1	2	3	1	1
FDAARGOS_716	3	5	5	1	2	2	1	**0**	1	2	3	1	1
FDAARGOS_690	3	5	5	1	2	2	1	**0**	1	2	3	1	1
M2901	3	5	7	1	2	2	1	**0**	1	2	3	1	1
2002017	3	5	8	1	2	3	1	**0**	1	2	3	1	1
G1663	3	4	8	2	3	2	1	**0**	1	3	3	1	0
1205	3	5	8	1	2	2	1	**0**	1	2	3	1	1
670	3	5	5	1	2	2	1	**0**	1	2	3	1	1
1602	3	5	8	1	2	3	1	**0**	1	2	3	1	1
74-1170	2	5	8	2	2	2	1	**0**	1	2	3	1	1
2016AM-0877	3	5	8	1	2	2	1	**0**	1	2	3	1	1
2013C-3749	3	5	8	1	2	3	1	**0**	1	2	3	1	1
AR-0425	3	5	8	1	1	2	1	**0**	1	2	3	1	1
AR-0424	3	5	7	1	2	2	1	**0**	1	2	3	1	1
AR-0423*	3	4	5	0	2	1	1	**0**	1	1	1	1	0
981*	3	4	6	0	2	1	1	**0**	1	1	1	1	0
439*	3	4	6	1	2	1	1	**0**	1	1	1	1	0
AUSMDU00008332	3	5	5	1	2	2	1	**0**	1	2	3	1	1
AUSMDU00010535	3	5	5	1	2	2	1	**0**	1	2	3	1	1
FDAARGOS_689*	2	3	8	1	2	3	1	**0**	1	1	3	0	0
301	3	4	8	1	2	1	1	1	1	3	3	1	1
M90T	5	4	5	1	3	2	1	1	1	2	2	1	0
NCTC 9728*	2	2	4	1	3	2	1	1	1	1	1	0	0
Y394	3	5	8	1	3	2	2	1	1	2	2	1	1
93-3063	3	5	8	1	2	2	1	1	1	2	2	1	1
94-3007	3	4	8	1	2	2	1	1	1	1	2	1	1
FDAARGOS_535	3	4	8	1	2	2	1	2	1	2	2	1	1
FDAARGOS_714*	2	1	11	1	**7**	**6**	**3**	2	1	**0**	3	1	1
64-5500*	1	1	6	0	**6**	**4**	**2**	2	1	**0**	2	1	1
71-2783*	2	3	6	1	2	2	1	2	1	1	1	1	0
89-141	3	5	7	1	3	5	1	1	1	2	2	**2**	0
AUSMDU00021847	3	5	8	1	**8**	2	1	1	1	2	2	**2**	0
AUSMDU00022017	3	5	8	1	**8**	2	1	1	1	3	2	**2**	0
83	3	5	8	1	**8**	2	1	1	1	3	2	**2**	0
FDAARGOS_74	3	4	9	1	**7**	1	1	1	1	2	2	**2**	0
FDAARGOS_713	3	4	9	1	**7**	1	1	1	1	3	2	**2**	0
