# Supplementary material for: Virulence plasmid pINV as a genetic signature for Shigella flexneri phylogeny
Source: Microb Genom. 2022 Jun 27;8(6):mgen000846. doi: 10.1099/mgen.0.000846 (PMC9455713; doi:10.1099/mgen.0.000846)
Supplement: Supplementary material 2 [file mgen-8-846-s002.pdf]

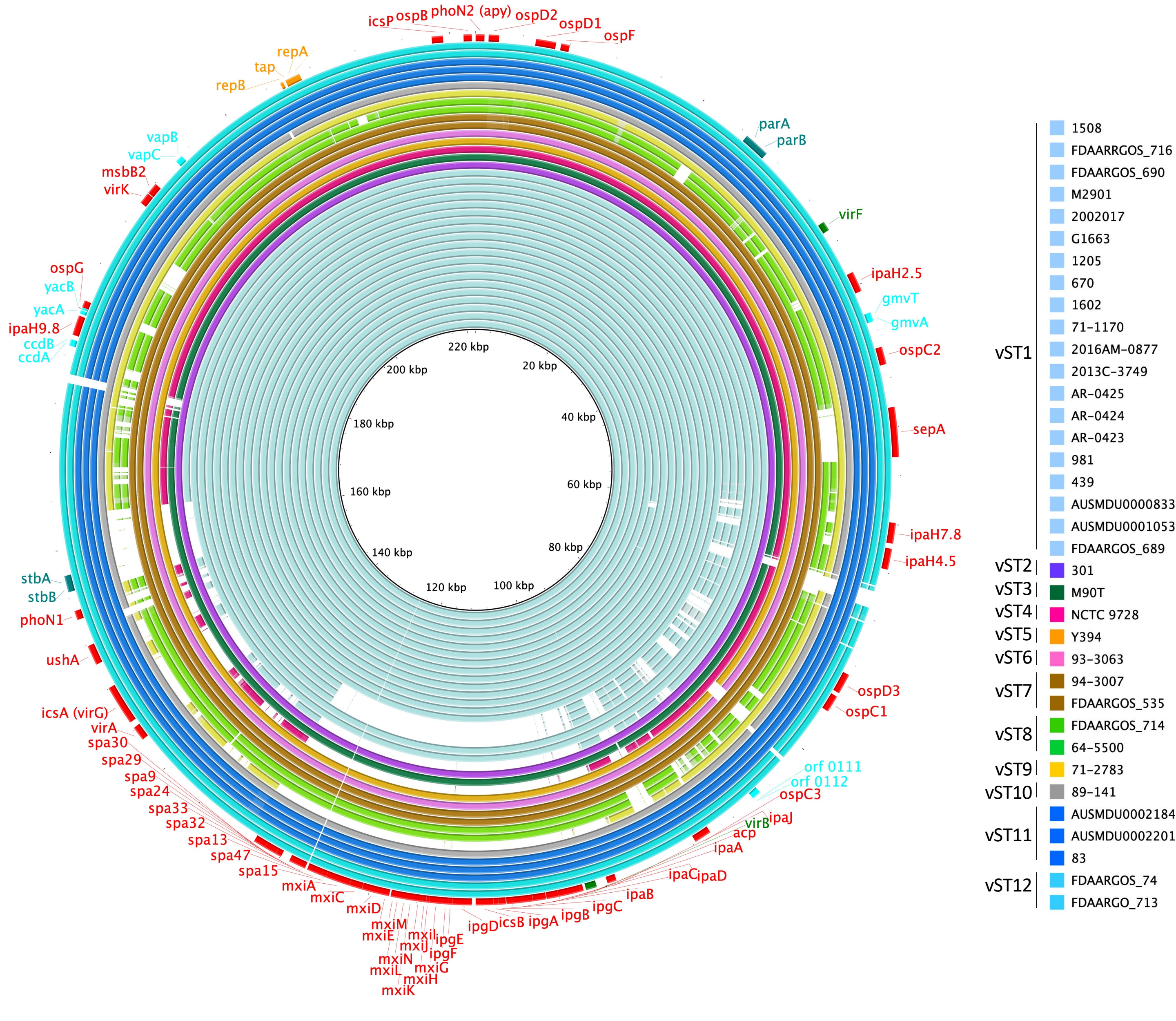
icsP
ospB
phoN2 (apy)
ospD2
ospD1
ospF
repA
tap
repB
parA
parB
vapB
vapC
msbB2
virK
virF
ipaH2.5
gmvT
gmvA
ospG
yacB
yacA
ipaH9.8
ccdB
ccdA
ospC2
sepA
ipaH7.8
ipaH4.5
stbA
stbB
phoN1
ushA
ospD3
ospC1
icsA (virG)
virA
spa30
spa29
spa9
spa24
spa33
spa32
spa13
spa47
spa15
mxiA
mxiC
mxiD
mxiM
mxiE
mxiI
mxiJ
mxiN
mxiL
mxiK
mxiH
mxiG
ipgF
ipgE
ipgD
icsB
ipgA
ipgB
ipgC
ipaB
ipaC
ipaD
ipaA
acp
virB
ipaJ
ospC3
orf 0111
orf 0112
220 kbp
20 kbp
200 kbp
40 kbp
180 kbp
60 kbp
160 kbp
80 kbp
140 kbp
120 kbp
100 kbp
vST1
1508
FDAARRGOS_716
FDAARGOS_690
M2901
2002017
G1663
1205
670
1602
71-1170
2016AM-0877
2013C-3749
AR-0425
AR-0424
AR-0423
981
439
AUSMDU0000833
AUSMDU0001053
FDAARGOS_689
vST2
301
vST3
M90T
vST4
NCTC 9728
vST5
Y394
vST6
93-3063
vST7
94-3007
FDAARGOS_535
vST8
FDAARGOS_714
64-5500
vST9
71-2783
vST10
89-141
vST11
AUSMDU0002184
AUSMDU0002201
83
vST12
FDAARGOS_74
FDAARGO_713
