# Supplementary figures and images for: Virulence plasmid pINV as a genetic signature for Shigella flexneri phylogeny
Source: Microb Genom. 2022 Jun 27;8(6):mgen000846. doi: 10.1099/mgen.0.000846 (PMC9455713; doi:10.1099/mgen.0.000846)

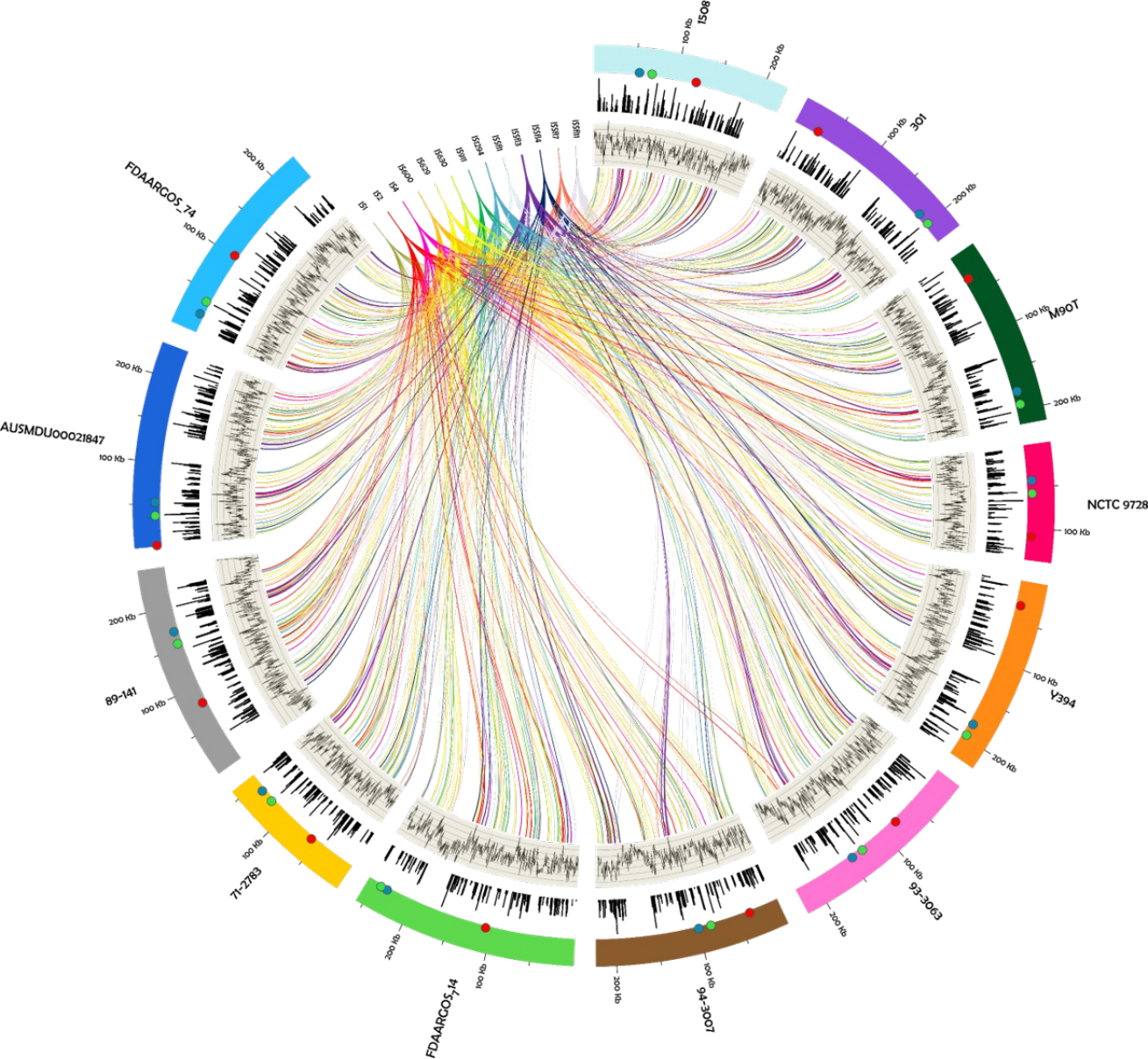

Supplement: Supplementary material 3 [file mgen-8-846-s003.pdf]
