# Supplementary material for: Virulence plasmid pINV as a genetic signature for Shigella flexneri phylogeny
Source: Microb Genom. 2022 Jun 27;8(6):mgen000846. doi: 10.1099/mgen.0.000846 (PMC9455713; doi:10.1099/mgen.0.000846)
Supplement: Supplementary material 4 [file mgen-8-846-s005.pdf]

Supplementary Figure 1. PCR amplification of *vapBC*, *yacAB* and *parAB* from *S. flexneri* 2a 2457T using primers indicated in Supplementary Table 2. W: water control. The sizes of a kb marker are shown.

Supplementary Figure 2. Virulence-associated genes, transcriptional regulators, partitioning systems, TA systems and replicon-associated genes on the alignment of pINV sequences from *S. flexneri* strains following vST grouping. Image created using BRIG 0.95 and BLASTN v2.2.29. *S. flexneri* 1508 pSF1508 (inner black ring) shown as the reference.

Supplementary Figure 3. Circos plot comparing the ISs found in the *S. flexneri* F27: A-: B- plasmid of the newly defined vST group 1 with the plasmids representing the other eleven groups.

From the outside to the inside of the figure can be found: the name of the strain or the IS just above the ideogram representing the plasmids or the IS; plasmids are labelled and colour-coded based on their belonging to the newly designated vST groups; the sequence position of the VST genes *parAB*, *yacAB* and *vapBC*, coloured in red, green and blue respectively; the CGs skew of the plasmids in windows of 500 bp; an histogram depicting the number of IS copies alongside the twelve plasmids; the links between the ISs and the plasmids.

Supplementary Table 1. List of *S. flexneri* assembled plasmid sequences downloaded from the Galata *et al*. plasmid database (1).

Supplementary Table 2. Parameters used to identify ISs on plasmid sequences.

Supplementary Table 3. List of 29 *S. flexneri* clinical strains arbitrarily selected from GenBank and *S. flexneri* 2457T strain used for an experimental test of vSTs. Identified vST groups, predicted serotypes and country of origin are indicated for each strain.

Supplementary Table 4. Comparison of the vST typing scheme with the single-linkage hierarchical clustering pipeline (pHierCC), implemented in the "cgMLST V1+HierCC V1" scheme at the EnteroBase database (https://enterobase.warwick.ac.uk/) on the analysis of the 68 *S. flexneri* strains studied in this work.

Supplementary Table 5. Number of copies of different ISs identified in *S. flexneri* pINVs. Asterisks indicate strains possessing pINVs with extended deletions. ISs present in consistently different numbers of copies in pINVs belonging to specific vST groups are highlighted in bold.

References:

1. Galata V, Fehlmann T, Backes C, Keller A. PLSDB: a resource of complete bacterial plasmids. Nucleic Acids Res. 2019;47(D1):D195-D202.
